# Three asymptomatic animal infection models of hemorrhagic fever with renal syndrome caused by hantaviruses

**DOI:** 10.1371/journal.pone.0216700

**Published:** 2019-05-10

**Authors:** Casey C. Perley, Rebecca L. Brocato, Steven A. Kwilas, Sharon Daye, Alicia Moreau, Donald K. Nichols, Kelly S. Wetzel, Joshua Shamblin, Jay W. Hooper

**Affiliations:** 1 Virology Division, United States Army Medical Research Institute of Infectious Diseases (USAMRIID), Ft. Detrick, Maryland, United States of America; 2 Pathology Division, United States Army Medical Research Institute of Infectious Diseases (USAMRIID), Ft. Detrick, Maryland, United States of America; 3 Molecular and Translational Sciences Division, United States Army Medical Research Institute of Infectious Diseases (USAMRIID), Ft. Detrick, Maryland, United States of America; University of Texas Medical Branch at Galveston, UNITED STATES

## Abstract

Hantaan virus (HTNV) and Puumala virus (PUUV) are rodent-borne hantaviruses that are the primary causes of hemorrhagic fever with renal syndrome (HFRS) in Europe and Asia. The development of well characterized animal models of HTNV and PUUV infection is critical for the evaluation and the potential licensure of HFRS vaccines and therapeutics. In this study we present three animal models of HTNV infection (hamster, ferret and marmoset), and two animal models of PUUV infection (hamster, ferret). Infection of hamsters with a ~3 times the infectious dose 99% (ID_99_) of HTNV by the intramuscular and ~1 ID_99_ of HTNV by the intranasal route leads to a persistent asymptomatic infection, characterized by sporadic viremia and high levels of viral genome in the lung, brain and kidney. In contrast, infection of hamsters with ~2 ID_99_ of PUUV by the intramuscular or ~1 ID_99_ of PUUV by the intranasal route leads to seroconversion with no detectable viremia, and a transient detection of viral genome. Infection of ferrets with a high dose of either HTNV or PUUV by the intramuscular route leads to seroconversion and gradual weight loss, though kidney function remained unimpaired and serum viremia and viral dissemination to organs was not detected. In marmosets a 1,000 PFU HTNV intramuscular challenge led to robust seroconversion and neutralizing antibody production. Similarly to the ferret model of HTNV infection, no renal impairment, serum viremia or viral dissemination to organs was detected in marmosets. This is the first report of hantavirus infection in ferrets and marmosets.

## Introduction

Hantaviruses are negative-sense RNA viruses transmitted to humans from small animal hosts. Different viral species are associated with one of two disease syndromes: hemorrhagic fever with renal syndrome (HFRS), or hantavirus pulmonary syndrome (HPS) [1]. Hantaan virus (HTNV), primarily found in Asia, is among the most prevalent HFRS-causing hantaviruses with a case fatality rate of between 1–15% [2]. Puumala virus (PUUV) causes most HFRS cases in Europe, though its case fatality rate is lower at <1% [3, 4]. There are currently no FDA licensed vaccines or therapeutics for either HFRS or HPS [5].

The Syrian hamster (*Mesocricetus auratus*) is the typical animal used to model hantavirus infection and disease. Andes virus (ANDV), an HPS-causing hantavirus, causes lethal disease in immunocompetent hamsters [6], while numerous other HPS-causing hantaviruses including Sin Nombre Virus (SNV) and Choclo virus cause lethal disease in hamsters immunosuppressed with dexamethasone and cyclophosphamide [7, 8]. In contrast to HPS-causing hantaviruses, exposure of hamsters to HFRS-causing hantaviruses such as HTNV, PUUV, Dobrava (DOBV) and Seoul (SEOV) leads to asymptomatic infection, despite viral dissemination, even when immunosuppressed (Hooper Lab, unpublished data) [8–11]. In these studies hamsters were exposed to high doses of HTNV and PUUV, far exceeding the infectious dose 99% (ID_99_) for the virus. Development and characterization of a uniformly infective, low-dose challenge model, enhances the hamster model’s usefulness in vaccine and therapeutic testing. In this report we present a low-dose hamster infection model for both HTNV and PUUV infected animals.

Ferrets (*Mustela putorius furo*) have become a popular animal model for a number of respiratory pathogens including influenza [12], coronavirus [13], Nipah virus [14], and morbillivirus [15], due to the similarity in lung physiology to humans. In addition, they have recently been described as a disease model of two hemorrhagic fever viruses, Bundibugyo virus and Ebola virus [16, 17], supporting viral replication without prior adaptation. Most hantavirus-related human disease occurs by aerosolized transmission of the virus from the excreta or secreta of infected rodents [18, 19], a model of viral infection for which the ferret is well suited. In this study we demonstrate that ferrets are capable of being infected by high titers of HTNV and PUUV, though aside from gradual weight loss infected animals exhibit no clinical symptoms or impaired renal function.

It has been established that infection of rhesus macaques (*Macaca mulatta)* with HFRS-causing hantaviruses (DOBV, SEOV, HTNV, and PUUV) leads to asymptomatic infection and seroconversion [9], while infection of cynomolgus macaques (*Macaca fascicularis)* with PUUV leads to a mild disease characterized by lethargy, mild proteinuria and hematuria, and kidney pathology, similar to mild HFRS in humans [20]. However, the macaques’ large size and cost limits their usefulness in therapeutic studies, especially when test article availability is limited, as is often the case in passive transfer studies. The common marmoset (*Callithrix jacchu*s) is becoming more popular for infectious disease studies. Its genetic similarity to humans, cost, relative safety, and small size make it an attractive alternative to traditional non-human primate species [21]. Marmosets have been used as a disease model for other viral agents including Dengue virus [22], Hepatitis C virus [23], influenza virus [24], Lassa fever virus [25], orthopox viruses [26–28], Rift Valley Fever virus [29], Eastern Equine Encephalitis virus [30], and filoviruses [31]. In this study we demonstrate that exposure of marmosets to HTNV leads to asymptomatic infection characterized by high levels of neutralizing antibodies. This is the first report of hantavirus infection in marmosets.

Medical countermeasures are products including biologics (e.g., vaccines and antibodies) and small molecule drugs that can be used to prevent or combat infectious disease outbreaks. This study presents three animal models of HTNV infection, and two models of PUUV infection that can be used to evaluate the efficacy of medical countermeasure that are intended to prevent or mitigate infection (e.g., vaccines) by these viruses through induction of sterile immunity.

## Materials and methods

### Viruses, cells and medium

HTNV strain 76–118 [32], PUUV strain K27 [33], and PUUV strains Beaumont, and Seloignes (gifts of Piet Maes, Leuven, Beligium) were propagated in Vero E6 cells (Vero C1008, ATCC CRL 1586) in T-150 flasks and cEMEM media (Eagle′s minimal essential medium with Earle′s salts (EMEM) containing 10% heat inactivated fetal bovine serum, 200 μM glutamine, 1% non-essential amino acids, 10 mM HEPES pH 7.4; and antibiotics [penicillin (100 U/ml), amphotericin B (250 μg/ml), and gentamicin (50 mg/ml)]. Virus was collected from infected-monolayer supernatants. Cell debris was removed by low speed centrifugation (2500 rpm in a table top centrifuge). HTNV and PUUV strain K27 were twice plaque purified according to published methods [6]. Virus stocks were aliquoted and stored at -60°C or colder. Virus identity has been confirmed by sequencing of the stocks.

### Ethics

Animal research was conducted under an IACUC approved protocol at USAMRIID (USDA Registration Number 51-F-00211728 & OLAW Assurance Number A3473-01) in compliance with the Animal Welfare Act and other federal statutes and regulations relating to animals and experiments involving animals. The facility where this research was conducted is fully accredited by the Association for Assessment and Accreditation of Laboratory Animal Care, International and adheres to principles stated in the Guide for the Care and Use of Laboratory Animals, National Research Council, 2011.

### Hamster procedures

Female Syrian hamsters 6–8 wks of age (Envigo, Indianapolis, IN) were anesthetized by inhalation of vaporized isoflurane using an IMPAC 6 veterinary anesthesia machine. Once anesthetized, animals were injected with the indicated concentration of virus diluted in PBS. Intramuscular (i.m.) injections (in the caudal thigh) consisted of 0.2 ml delivered with a 1ml syringe with a 25-gauge, 5/8 in needle. Intranasal (i.n.) instillation consisted of 50 μl total volume delivered as 25 μl per nare with a plastic pipette tip. Blood sampling from the vena cava occurred under previously stated methods of anesthesia, and was limited to 7% of a hamster’s total blood volume per week. At time of arrival animals were randomized into experimental groups. Animals were housed in small animal pans, not exceeding four animals to a pan, in a climate and humidity controlled animal biosafety level 3 (ABSL-3) with a 12-hour light/dark cycle. Animals had pelleted food and water provided *ad libitum*. Enrichment in the form of toys, nesting material and supplemental treats was provided. Humane endpoint conditions were established as decreased mobility (inability to obtain food and water) and subdued response to stimulation, and animals were monitored daily during the experiment. As infected animals did not become ill, these criteria were not met and animals were euthanized by terminal blood collection from the heart after administration of Ketamine–acepromazine–xylazine (KAX)(USAMRIID, Fort Detrick MD) and prior to intracardiac injection with pentobarbital sodium (USAMRIID) at the end of the study. Due to lack of illness no pain relief, aside from anesthesia during procedures, was required.

### Hamster sample size justification

Groups of 10 hamsters were used to determine the infectious dose 50% (ID_50_) and 99% (ID_99_). Numerical simulations using SAS Probit indicate that a minimum of 3 groups spanning infection rates 0–100% with 10 animals per group yield stable estimates of the values with confidence intervals reaching out to 0.7log_10_ (approximately 5-fold) under monotome assumptions of response profiles for intermediate doses. As our initial selection of dosages did not meet the desired infection rate span, some dosage groups were repeated leading to 20 hamsters per group. In the serial euthanasia study three hamsters per group were used. This is the minimum number required to provide collection of sufficient samples for detection of antibodies and viral kinetics in tissues. For each experiment pre-sera from animals served as negative control.

### Ferret procedures

Adult, female neutered and descented ferrets (Marshall Farms, North Rose, NY), were anesthetized by inhalation of vaporized isoflurane using an IMPAC 6 veterinary anesthesia machine, or i.m. injection of Telazol (Zoetis, Parsippany, NJ). Injections (i.m. and i.n.) and blood sampling were conducted under the same condition as hamsters. Intraperitoneal (i.p.) injections consisted of 1 ml delivered with a 3 mL syringe and a 23-guage needle. Microchips (BMDS, Seaford, DE) were used to identify and ascertain temperature during ferret experiments. In the first ferret challenge study faulty chips lead to inaccurate temperature readings and were only used for identification purposes. Animals were randomized upon receipt into experimental groups. Ferrets were socially housed in metal caging, two to a cage, with sight lines to additional animals in the study, in a climate and humidity controlled ABSL-3 with 12/hour light and dark cycles. Each cage had a nesting box with bedding material, and numerous tubes and shelfs for play. Ferrets had access to pelleted food supplemented with treats and potable water, through an automated watering system. Enrichment in the form of manipulada (tubes, balls, mirrors) and food was provided. Animals were observed daily by trained personnel in addition to general husbandry assessments. Humane euthanasia criteria, defined as both dyspnea, loss of mobility (to obtain food and water) and >20% weight loss. At the end of the experiment, terminal blood samples were collected from the heart after administration of KAX and prior to intracardiac injection with pentobarbital sodium. No pain relief, aside from anesthesia during procedures, was used.

### Ferret sample size justification

When disease occurs independently in each of four ferrets with 50% probability, the experiment will have odds about 9:1 in favor of producing at least one diseased ferret. Conversely failure to observe any diseased ferret in a group of four will yield a 95% confidence interval extending from 0–50%. That is, with 95% confidence it will be admitted that the true disease rate may be 50% or less. For this reason groups of four ferrets were used for the experiments. For each experiment pre-sera from animals served as a negative control.

### Marmoset procedures

Adult marmosets weighing over 300g were anesthetized by inhalation of vaporized isoflurane using an IMPAC 6 veterinary anesthesia machine. Once anesthetized, animals were injected with the indicated concentration of virus diluted in PBS. I.m. injections (in the caudal thigh) consisted of 0.2 ml delivered with a 1ml syringe with a 25-gauge, 5/8 in needle. Blood sampling from a femoral vein occurred under previously stated methods of anesthesia, and were limited to 7% of each marmoset’s total blood volume per week. At time of euthanasia, terminal blood samples were collected from the heart after anesthetization by i.m. injection of Telazol and prior to intracardiac administration of pentobarbital sodium. Animals were housed in containment as previously described [26]. In brief, animals were singly housed in metal cages meeting current standards in a climate and humidity controlled room. Animals were fed pelleted food supplemented with fruits and treats daily, and were provided potable water through an automatic watering system *ad libitum*. Enrichment in the form of manipulada (i.e. toys, metal mirrors), foraging devices, treats, and fruit were provided daily. Animals were observed daily by trained personnel in addition to general husbandry assessments. Animals were observed daily by trained personnel in addition to general husbandry assessments. Animals found moribund (defined as labored breathing, decreased food consumption, persistent prostration and moderate unresponsiveness) would be euthanized under humane endpoint criteria, however, as animals did not become ill during the study this criteria was not met. No additional pain relief, aside from anesthesia during procedures, was necessary. All work was performed in an ABSL-3 laboratory.

### Marmoset sample size justification

The marmoset study requires a sample size of 3 for adequate power to determine if the incidence of seroconversion is significantly greater than that which would be expected in the population. This sample size will allow the experimenter to detect seroconversion in at least 2 of 3 animals (66%) versus the expected population constant of <1% at a 95% confidence level using a one-tailed binomial test for proportions.

### ELISA

The enzyme-linked immunosorbent assay (ELISA) used to detect nucleocapsid protein (N) specific antibodies (N-ELISA) was described previously [34]. Species-specific secondary antibodies were used at the following concentrations: peroxide-labeled anti-hamster (1:10,000) (Sera Care, Gaithersburg, MD), peroxide-labeled anti-ferret (1:5,000) (Sigma Aldrich, St. Louis, MO), and alkaline phosphatase conjugated anti-monkey (1:1,000) (MilliporeSigma, St. Louis, MO). Assays using peroxide labeled antibodies were developed with TMB microwell peroxidase substrate (Sera Care) at an absorbance of 450 nm; assays using alkaline phosphatase conjugated antibodies were developed with p-nitrophenyl phosphate (PNPP) (ThermoFisher Scientific, Waltham, MA) at 405 nm. A sample was considered positive if its peak optical density (OD) value was greater than either 0.025 or the background value (the average of three negative controls + 3 times their standard deviation), whichever was higher. The specific OD sum is the summation of all values greater than background and represents the area under the titer curve.

### Plaque Reduction Neutralization test (PRNT)

PRNT assays were performed as previously described with minor modifications [35–37]. HTNV-infected monolayers were fixed 7 days post-infection, while PUUV-infected monolayers were fixed 10 days post infection by 2 mL of 10% formalin per well. Immunostaining was performed as previously described [38]. All sera samples were assayed in duplicate beginning at a 1:20 final dilution. PRNT_50_ values represent the reciprocal dilution at which the serum neutralizes 50% of the virus.

### Isolation of RNA and real time RT-PCR

Approximately 200 mg of organ tissue was homogenized in 1.0 mL of TRIzol (ThermoFisher, Waltham, MA) reagent using M tubes on the gentleMACS (Miltenyi Biotec,Auburn, CA) dissociation system on the RNA setting. RNA was extracted from TRIzol per manufacturer’s protocol. A Nanodrop 8000 was used to determined RNA concentration, which was then raised to either 100 ng/μL or 1,000 ng/μL in UltraPure distilled water (Thermofisher). Real-time PCR was conducted on a BioRad CFX thermal cycler using either an Invitrogen Power SYBR Green RNA-to-Ct one-step kit (Thermofisher) or Brilliant II QRT-PCR 1 -Step Master Mix (Agilent, Santa Clara, CA) according to the manufacturer’s protocols. For spiked assays the master mix was spiked with either HTNV or PUUV viral RNA prior to addition to the samples. For HTNV, primer sequences were 594F 5'-AAG CAT GAA GGC AGA AGA GAT -3' and 830R 5'-TAG TCC CTG TTT GTT GCA GG-3'. Cycling conditions were 30 min at 48°C, 10 min at 95°C, followed by 40 cycles of 15 sec at 95°C and 45 sec at 60°C. Data acquisition occurs following the annealing step [39]. For PUUV, primer sequences were 181F 5'-AGG CAA CAA ACA GTG TCA GCA-3' and 334R 5'-GCA TTC ACA TCA AGG ACA TTT CCA TA-3' with a FAM-conjugated probe 278 5’ 5'-6-FAM-CTG ACC CGA CTG GGA TTG AAC CTG ATG-BHQ-1-3'. Cycling conditions were 30 min at 48°C, 10 min at 95°C, followed by 40 cycles of 15 sec at 95°C and 1 min at 60°C [40].

### Cell culture amplification of infectious virus from urine

T-25 flasks of one week old Vero E6 cells were infected with 50 μL of urine plus an additional 450 μL of cEMEM media. After a 1 hr adsorption at 37°C with 5% CO_2_, the volume was raised to 3.5 mL. On Day 4 post infection supernatant was collected and frozen down, 500 μL of which was used to infected fresh Vero E6 cells at a later time point. After a 1 hr adsorption at 37°C with 5% CO_2_, the volume was raised to 3.5 mL. On days 7, 11, 14, 17, 21 and 28 1.2 mL of culture supernatant was collected and frozen down. The volume of cEMEM in the flask was raised to 3.5 mL with fresh media.

### Plaque assay

Approximately 200 μg of organ tissue were homogenized in 1 mL of cEMEM media using M tubes on the gentleMACS dissociation system on the RNA setting. Plaque assays using urine, sera, or organ homogenate were then performed beginning at the 1:10 dilution as described in [6, 41] with minor modifications. For spiked plaque assays the protocol was identical except for equivalent amounts of virus being spiked into either media alone (control), or the 1:10–1:1,000 dilution of organ homogenate. HTNV-infected monolayers were fixed 7 days post-infection, while PUUV-infected monolayers were fixed 10 days post infection by 2 mL of 10% formalin per well. Immunostaining was performed as previously described [38].

### Post mortem procedures

Following euthanasia, necropsies were performed. Samples were collected aseptically for the virology studies described above. For the hamsters and ferrets, samples of the following were collected: heart, lung, liver, spleen, kidney, brain, and urine. In addition, ferrets had samples of intestine, adrenal gland, pituitary gland, and eye were collected. Samples of the following were collected from the marmosets: heart, lung, liver, spleen, kidney, intestine, and brain. After the virology samples had been collected, all major internal organs in each animal were also sampled for histology.

### Preparation of tissues for histology and immunohistochemistry

Tissues were fixed in 10% neutral-buffered formalin for ≥21 days. Tissues were then trimmed, processed under vacuum through increasing concentrations of alcohols, and embedded in paraffin. Paraffin embedded tissue sections of 5–6 μm were cut and mounted on glass slides, stained with hematoxylin and eosin (H&E), and mounted under a glass coverslip for routine histologic evaluation. The paraffin-embedded tissues used for producing the H&E-stained histology slides were also utilized for immunohistochemistry (IHC) studies in the hamsters. Immunolocalization of HTNV in tissues was performed with an immunoperoxidase procedure (horseradish peroxidase EnVision system; Dako) according to the manufacturer’s directions. The primary antibody was an anti-HTNV nucleocapsid rabbit polyclonal antibody diluted 1:3,500 (ferret) or 1:5,000 (hamster) (BEI Resources, Manassas, VA). After deparaffinization and peroxidase blocking, tissue sections were pretreated with proteinase K for 6 min at room temperature, rinsed, and then covered with primary antibody and incubated at room temperature for 30 min. They were rinsed, and then the peroxidase-labeled polymer (secondary antibody) was applied for 30 min. Slides were rinsed, and a substrate-chromogen solution (3,3’-diaminobenzidine; Dako, Santa Clara, CA) was applied for 5 min. The substrate-chromogen solution was rinsed off the slides, and the slides were stained with hematoxylin and rinsed. The sections were dehydrated and cleared with xyless, and then a coverslip was placed.

### Immunosuppression with cyclophosphamide (Cyp)

On the indicated days, anesthetized ferrets were injected i.p. with water soluble Cyp (Baxter Health Care Corporation, Deerfield, IL) with the indicated dosages per body weight of drug diluted in sterile phosphate-buffered saline (PBS), pH 7.4. In the first experiment ferrets were administered a loading dose of 30 mg/kg on Day 41 post infection, with maintenance doses of 30 mg/kg administered every other day until euthanasia. In the second experiment, ferrets were administered a loading dose of 30 mg/kg on Day -1, and a maintenance dose of 10 mg/kg on Day 1, 3, 11, and 13. Administration of Cyp was discontinued between days 3 and 11 due to secondary infection. To combat the infection (rapid onset of fever and weight loss), ferrets were treated with 5mg/kg i.m. enrofloxicin(Norbrook Laboratories, Overland Park, KS) twice daily per veterinarian instructions starting on Day 4. Beginning on Day 11 with the resumption of immunosuppression ferrets were treated prophylactically with 10 mg/kg i.m. enrofloxicin once daily).

### Hematology

Blood samples of 0.5 mL were collected in lithium heparin capillary blood collection tubes and analyzed using an Advia 120 hematology analyzer (Software version 3.1.8.0-MS). Per manufacturer’s recommendation, the dog setting was used for the complete blood count (CBC) and the guinea pig setting was used for the white blood cell differential (WBC) in hamsters. For ferrets, the dog setting was used for both the CBC and WBC.

### Pseudovirion neutralization assay (PsVNA)

The PsVNA used to detect neutralizing antibodies in sera was described previously [42, 43]. This is a replication-restricted, recombinant vesicular stomatitis virus (rVSV*ΔG) expressing luciferase, which is pseudotyped with the Hantaan glycoprotein. First, heat-inactivated sera was diluted 1:10, followed by five-fold serial dilutions that were mixed with an equal volume of Eagle′s minimum essential medium with Earle′s salts and 10% fetal bovine sera containing 4000 fluorescent focus units of Hantaan pseudovirions. This mixture was incubated overnight at 4°C. Following this incubation, 50 μl was inoculated onto Vero cell monolayers in a clear bottom, black-walled 96-well plate in duplicate. Plates were incubated at 37°C for 18–24 hr. The media was discarded and cells were lysed according to the luciferase kit protocol (Promega, Madison, WI). A Tecan M200 Pro was used to acquire luciferase data. The values were graphed using GraphPad Prism (version 7) and used to calculate the percent neutralization normalized to cells alone and pseudovirions alone as the minimum and maximum signals, respectively. The percent neutralization values for duplicate serial dilutions were plotted. Fifty percent PsVNA (PsVNA_50_) titers were interpolated from 4-parameter curves, and geometric mean titers were calculated.

### Blood chemistry

Blood was collected in serum separator tube, and spun at 500x g to isolate sera. Ferret sera was analyzed on the Piccolo comprehensive metabolic panel, and marmoset sera was analyzed on the Piccolo general chemistry 13 panel per manufacturer’s instructions (Abaxis Global Diagnostics, Union City, CA).

### Urinalysis

Urine was expressed from anesthetized ferrets and analyzed by urinalysis regent strips (VWR, Radnor, PA).

### Statistical analysis

A Bayesian probit model was used to estimate 95% highest posterior density intervals for a 50% and 95% infectious dose calculation. Student’s t-test and Mann-Whitney tests were used to compare white blood cell levels pre- and post-Cyp administration. P-values of <0.05 were considered significant. Standard deviation of data was assessed to ensure data was normally distributed prior to use of Student’s t-test. Analyses were conducted using GraphPad Prism (version 7); Bayesian analyses were performed using SAS.

## Results

### Calculation of ID_50_ and ID_99_ for hamster model of HTNV and PUUV infection

**Fig 1:**
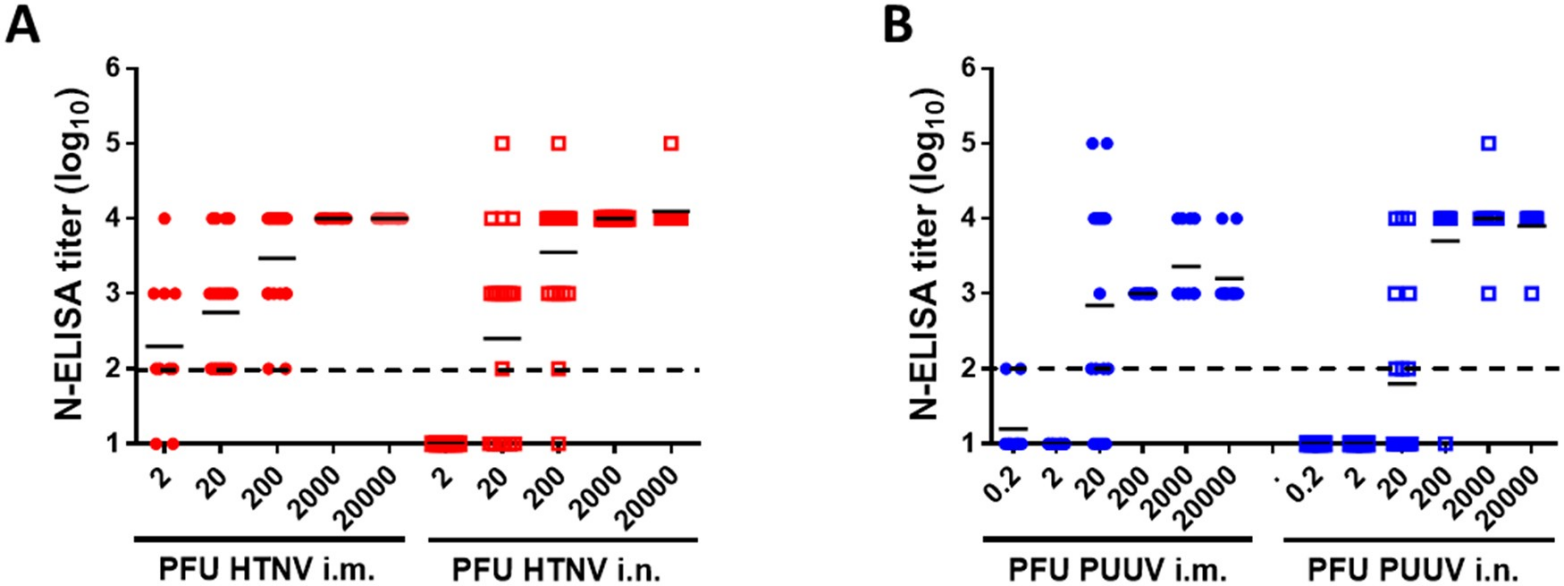
HTNV and PUUV infect hamsters in a dose dependent manner. Syrian hamsters were challenged with varying concentrations of either HTNV (**A**) or PUUV (**B**) through the i.m. (left) or i.n. (right) route. Between 28 and 35 days post infection hamsters were terminally bled and N-ELISA endpoint titers (log_10_) were used to determine infection status. The mean titer is displayed for each group, and the limit of detection (2log_10_) is depicted as a dashed line.

We have previously demonstrated that Syrian hamsters are capable of being infected by HFRS-causing hantaviruses, but they do not develop any signs of clinical disease [9]. To develop standard models of HTNV and PUUV infection for future evaluation of vaccines and medical countermeasures, groups of between 10 and 20 hamsters were exposed to serial ten-fold dilutions of either HTNV or PUUV by either the i.m. or i.n. route (from 2–20,000 PFU HTNV or 0.2–20,000 PFU PUUV). Between 28–35 days post infection, hamsters were terminally bled with infection status monitored by N-ELISA titers (Fig 1). From these data the doses required to infect 50% (ID_50_) and 99% (ID_99_) were calculated (Table 1).

**Table 1:** ID_50_ and ID_99_ for HTNV and PUUV.

Virus	Infection Route	ID_50_	ID_99_
HTNV	i.m.	1.5	3.4
	i.n.	17.7	346
PUUV	i.m.	6.0	832
	i.n.	30.7	637

### Refinement of low-dose HTNV and PUUV infection hamster models

To further characterize a low-dose standard hamster infection model for HFRS-causing hantaviruses a hamster serial sacrifice study was performed. Hamsters were challenged with either 10 PFU (~3 ID_99_) HTNV i.m., 500 PFU (~1.5 ID_99_) HTNV i.n., 1,000 PFU (~1 ID_99_) PUUV i.m., or 1,000 PFU (~1.5 ID_99_) PUUV i.n. On various days post infection, groups of three hamsters were euthanized to monitor viral and serological parameters.

**Fig 2:**
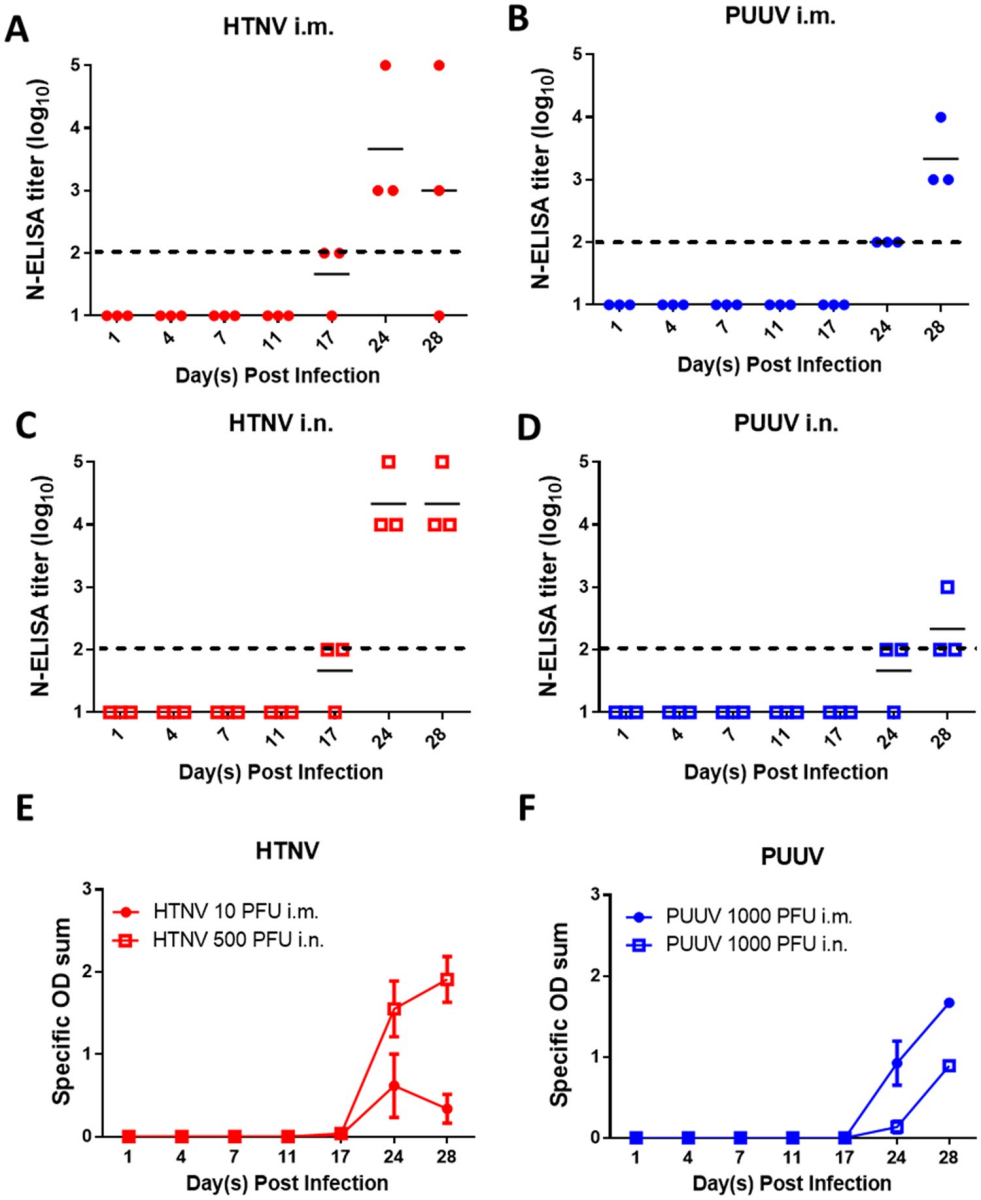
Kinetics of seroconversion in low dose hamster models. Syrian hamsters were infected either 10 PFU HTNV i.m., 500 PFU HTNV i.n., 1000 PFU PUUV i.m., or 1000 PFU PUUV i.n. Hamsters were terminally bled at various points post infection and N-ELISA endpoint titers (log_10_) were used to determine infection status. The mean titer (A-D), and specific OD sum ± standard error of the mean (SEM) (E-F) for the N-ELISA is displayed for each group. The limit of detection (2log_10_) for the N-ELISA is depicted as a dashed line. The specific OD sum represents the area under the titer curve.

Seroconversion occurred, at least partially, by Day 17 post HTNV infection and Day 24 post PUUV infection. Seroconversion on Day 28 post HTNV i.m. infection was incomplete, though viral genome was recovered from all hamsters euthanized that day indicating a productive HTNV infection occurred (Fig 2). To confirm seroconversion, all hamsters euthanized on days 17, 24, and 28 were assayed for neutralizing antibodies by PRNT regardless of N-ELISA seroconversion status. All HTNV infected hamsters with N-ELISA titers had neutralizing antibodies as measured by PRNT_50_, with all but one having full neutralization of virus at a 1:20 dilution of sera. (S1A and S1B Fig). Similarly, all PUUV hamsters with N-ELISA titers had neutralizing antibodies as measured by PRNT_50_, though three of the five PUUV i.n. challenged hamsters did not have complete neutralization of the virus at a 1:20 dilution of sera (S1C and S1D Fig). Two of three PUUV i.m. challenged hamsters that had not seroconverted on Day 17 post infection had low levels of neutralizing antibodies, while none of the PUUV i.n. challenged hamsters that were seronegative by N-ELISA had neutralizing antibodies. For both HTNV and PUUV infected hamsters, infection via the i.m. route lead to a more robust neutralizing antibody response than then i.n.route.

**Fig 3:**
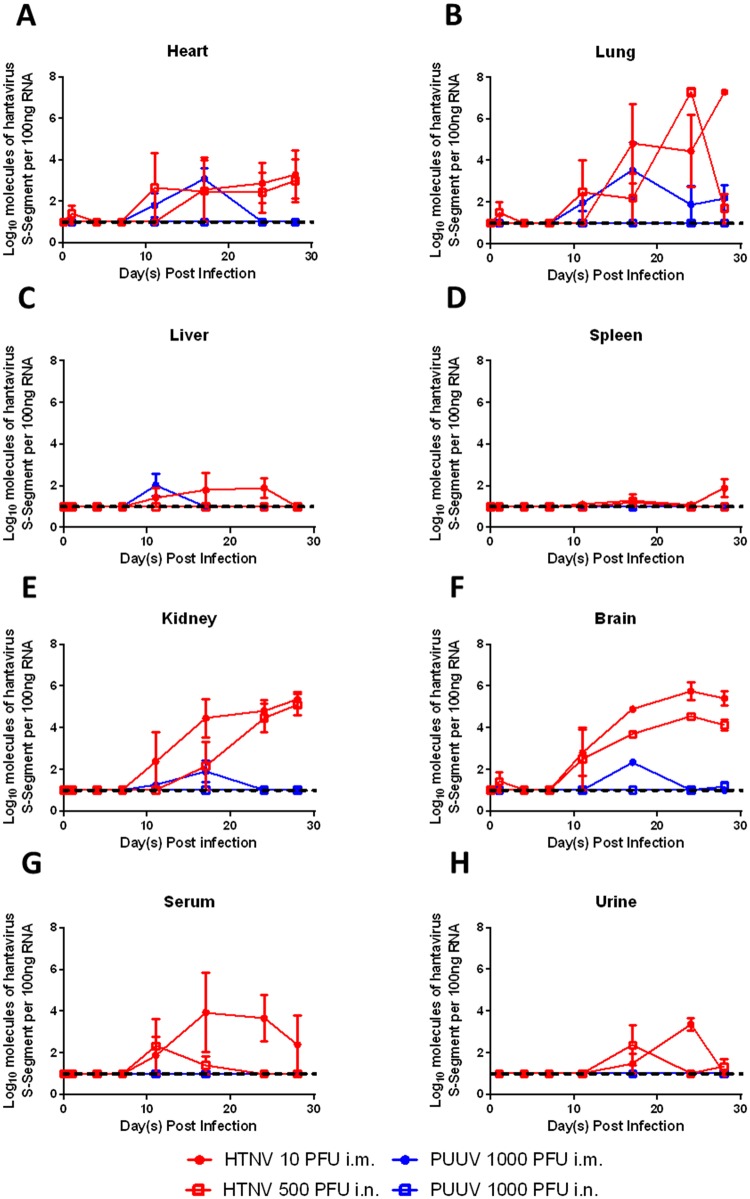
Viral genome is detectable in the organs and sera of infected hamsters. Syrian hamsters were infected either 10 PFU HTNV i.m., 500 PFU HTNV i.n., 1000 PFU PUUV i.m., or 1000 PFU PUUV i.n. Hamsters were euthanized at various points post infection and heart (**A**), lung (**B**), liver (**C**), spleen (**D**), kidney (**E**), brain (**F**), sera (**G**), and urine (**H**) were evaluated by RT-PCR for the presence of viral genome. The mean ± SEM is displayed at each time point. The dashed line indicates the limit of detection (1log_10_).

**Fig 4:**
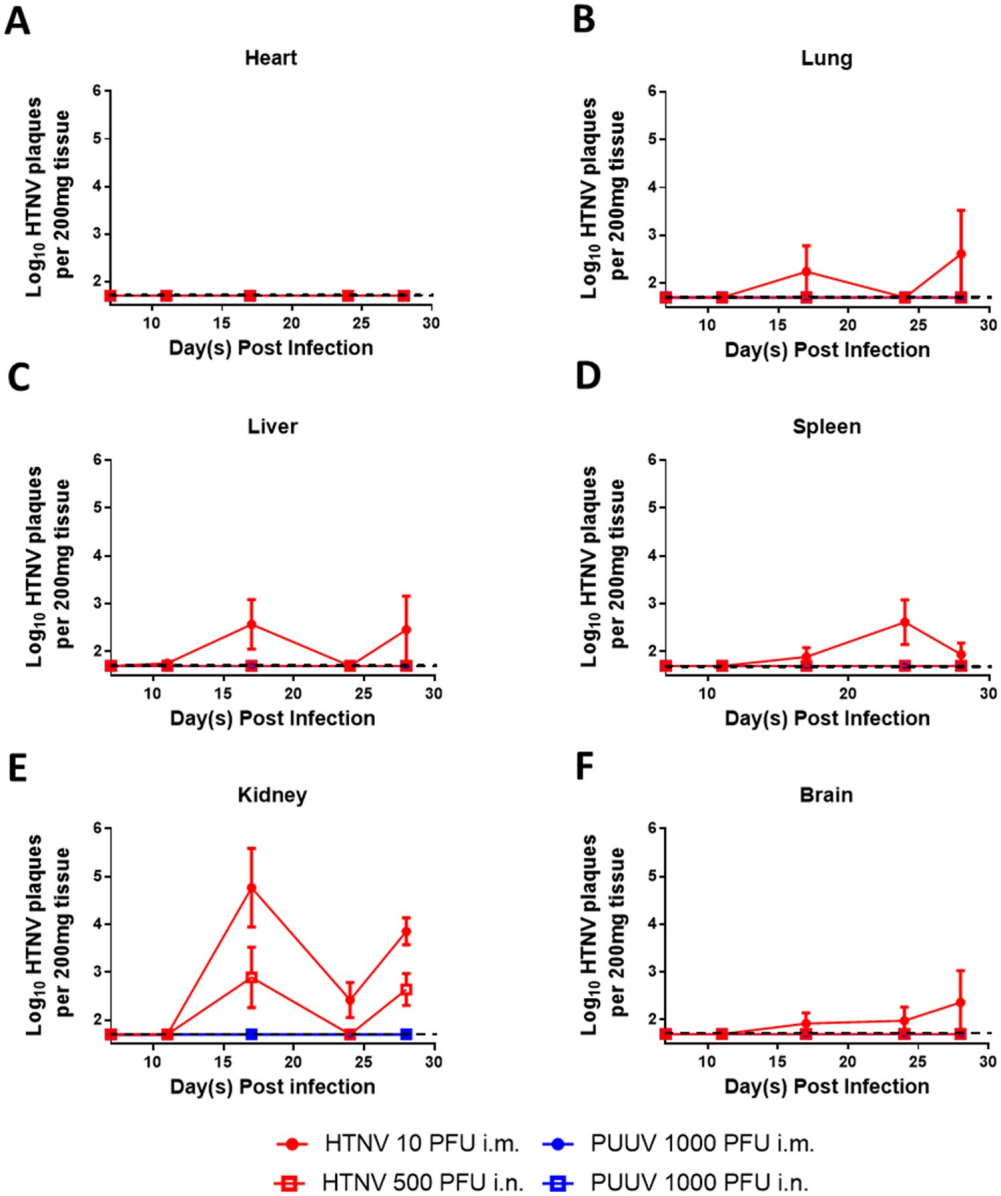
Infectious virus was isolated from the organs of HTNV i.m. infected animals. Syrian golden hamsters were infected with 10 PFU HTNV i.m. and sacrificed at various time points post infection. Heart (**A**), lung (**B**), liver (**C**), spleen (**D**), kidney (**E**), and brain (**F**) from the time range where the organ was RT-PCR positive were sampled by plaque assay for the presence of infectious virus. The mean ± SEM is displayed at each time point. The limit of detection (1.7 log_10_) is depicted as a dashed line.

The kinetics of HTNV and PUUV infection in the heart, lung, liver, spleen, kidney, and brain were monitored by both RT-PCR and plaque assay (Figs 3 and 4). HTNV infected by either the i.m. or i.n. route resulted in a persistent infection. High levels of viral genome were detected in the heart, lung, kidney and brain of HTNV infected hamsters beginning on either Day 11 or 17 post infection (Fig 3A, 3B, 3E and 3F). Infection of the kidney and brain was found in all examined hamsters beginning on either Day 17 or 24 post infection, while the high titers of viral genome detected in the heart and lung were present in only one or two hamsters at each time point. Low levels of viral genome were detected between days 11 and 24 post infection in the liver of HTNV i.m. but not i.n. infected hamsters (Fig 3C). Hardly any viral genome was detected in the spleen (Fig 3D). To confirm the lack of viral genome in the spleen was not due to the presence of inhibitors all spleen samples from i.m. and i.n. infected hamsters were spiked with HTNV prior to RT-PCR. No significant inhibition of the spiked RNA was noted, indicating that HTNV infection does not result in virus dissemination to the spleen (S2A and S2B Fig).

The detection of PUUV in organs was transient after i.m. infection, occurring between Day 11–17 for the heart, liver, kidney and brain, with no virus detected in the spleen (Fig 3A, 3C, 3E and 3F). Viral genome was more persistent in the lung where it was detected in at least one of three hamsters on/after Day 11 post infection (Fig 3B). A small amount of viral genome detected in the brain of a PUUV i.n. infected hamster, 28 days post challenge, is the only viral genome detected in any organ at any time point post PUUV i.n. infection (Fig 3F).

Serum viremia was detected in hamsters challenged with HTNV i.m. between 11 and 28 days post infection, though the presence of virus was sporadic except for Day 24. Serum viremia was only detected in two hamsters challenged with HTNV i.n., one on Day 11 and one on Day 17. No serum viremia was detected in hamsters challenged with PUUV by either route (Fig 3G). Similarly, the presence of viral genome was sporadically detected in the urine of HTNV infected hamsters between days 17–28, but was not detected in PUUV challenged hamsters (Fig 3H).

In HTNV i.m. infected hamsters, infectious virus was first detected in the liver beginning at Day 11, and in the lung, liver, spleen, and kidney at Day 17 post infection (Fig 4B, 4C, 4D and 4E). With the exception of the kidney, in which infectious virus was recovered from every hamster after 17 days post infection, infectious virus was recovered from the lung, liver, brain and spleen in only a portion of hamsters at each time point. No infectious virus was detected in the heart at any time point (Fig 4A). Virus recovery from HTNV i.n. infected hamsters was markedly lower, with infectious virus being detected only in the kidney on Day17 and 28 post infection (Fig 4E). No infectious virus was recovered from the organ of any PUUV infected hamster (Fig 4).

To confirm that the lack of infectious virus in the spleen was not due to the presence of inhibitors select samples were spiked with HTNV virus prior to a plaque assay. No significant inhibition of the spiked RNA was noted, confirming the RT-PCR results that that HTNV infection does not result in viral dissemination to the spleen (S2C and S2D Fig).

No infectious virus was detected in the urine for any hamster tested, even those that were RT-PCR positive for HTNV viral genome. In human ANDV infected patients with ANDV antigen positive urine, samples had to be cultured in Vero E6 cells for between 16–22 days post infection before infectious virus was detected [44]. Three HTNV RT-PCR positive and three HTNV RT-PCR negative urine samples were cultured in Vero E6 cells, with supernatant collected on various days post infection for evaluation by RT-PCR (S3A Fig). All samples were HTNV RT-PCR negative for virus in the supernatant of infected cells at Day 4 post infection, the first time point examined. All three RT-PCR positive urine samples yielded infectious virus (two on Day 15, one on Day 32), while two of the RT-PCR negative urine samples also yielded infectious virus (on Day 18) (S3B Fig) as measured by the conversion of cell supernatant to HTNV RT-PCR positive. All samples were evaluated by plaque assay on Day 32 post infection. These findings were confirmed, all samples whose cell supernatant became RT-PCR positive post infection were positive for infectious virus by plaque assay. The one sample whose cell supernatant did not convert to RT-PCR positive, remained negative for infectious virus by plaque assay (S3C Fig).

Patients infected with HFRS hantaviruses exhibit leukocytosis and thrombocytopenia during infection [45, 46]. At every time point post infection, EDTA-treated whole blood from HTNV and PUUV exposed hamsters was evaluated to determine if changes in white blood cell count or platelets occurred. No changes were observed (S4 Fig).

To further characterize disease, tissue sections from HTNV i.m. infected hamsters were analyzed by IHC and H&E to assess viral localization and any pathologic changes (S5 Fig). No significant histopathological findings were noted in the kidney or brain. Splenic follicular lymphoid hyperplasia and hepatic extramedullary hematopoiesis in the liver were each noted in three hamsters. Both are seen in animals from later time points in the study (days 17–28) and likely represent a reaction to HTNV infection, though other unidentified antigenic stimuli cannot be ruled out. Five hamsters between days 4 and 17 post infection exhibited a minimal to mild inflammation of the pericardium, characterized by a mixed lymphoplasmacytic histiocytic and neutrophilic infiltrate. Occasional macrophages in the inflammatory infiltrate within the pericardium are immunopositive suggesting a possible association with HTNV; however, no evidence of cardiac tissue injury is associated with the presence of HTNV antigen. Beginning on Day 4 post infection 66% (12/18) hamsters exhibit minimal (likely subclinical) respiratory lesions consisting of interstitial neutrophilic and histiocytic infiltrates in the lungs, with 44% (8/18) also exhibiting minimal amounts of alveolar edema. An additional hamster had minimal alveolar edema but not pulmonary infiltrates. Such findings suggest a response to antigenic stimulus and the presence of immunopositive endothelial cells, pneumocytes and alveolar macrophages suggest a response to HTNV infection.

Immunohistochemistry staining was uniformly negative in all tissues on Day 1 post challenge. Between Day 4 and 11 post challenge minimal antigen was sporadically detected in macrophages, pneumocytes and endothelial cells. By Day 17 post challenge mild to moderate levels of antigen were observed in endothelial cells and choroid ependymal cells in the brain, in endothelial cells, macrophages and pneumocytes in the lung, and endothelial cells in the kidney. Minimal to mild levels of immunopositivity were found in endothelial cells and macrophages in the heart, and in endothelial cells, hepatocytes and Kupffer cells in the liver. Surprisingly, given the lack of viral genome and infectious virus recovered, mild to moderate amounts of immunopositive macrophages, lymphocytes and endothelial cells were found in the spleen (Table 2, S5 Fig).

**Table 2:** Pathology scores for HTNV IM infected hamsters.

Day	Hamster #	Brain	Heart	Lung	Liver	Spleen	Kidney
0	Pre	0	0	0	0	0	0
	Pre	0	0	0	0	0	0
	Pre	0	0	0	0	0	0
1	801	0	0	0	0	0	0
	802	0	0	0	0	0	0
	803	0	0	0	0	0	0
4	804	0	0	0	0	0	0
	805	0	1+/m	0	0	0	0
	806	0	0	0	0	0	0
7	807	0	0	0	0	1+/m	0
	808	0	0	0	0	0	0
	809	1+/e	0	0	1+/e	0	0
11	810	2+/e	0	2+/e,p	0	1+/m	2+/e
	811	0	0	0	0	0	0
	812	1+/e	1+/m	0	0	0	0
17	813	3+/e	0	2+/e,p	2+/e,h	2+/m	2+/e
	814	2+/e	0	4+/e,p	1+/h,k	2+/m	2+/e
	815	2+/e	1+/e	3+/e,p	2+/e,k	1+/m	2+/e
24	816	2+/e	1+/e	3+/e,p	1+/k	2+/m	2+/e
	817	2+/e,c	1+/e	2+/e,m,p	1+/e,h	3+/m,l	2+/e
	818	3+/c,e	1+/e	3+/e,m,p	1+/e,k	3+/m,l	3+/e
28	819	3+/c,e	0	3+/e,m,p	1+/k	1+/e	2+/e
	820	3+/c,e	0	3+/e,m,p	1+/e,k	0	3+/e
	821	2+/e,c	1+/e	3+/e,m,p	1+/e,h,k	2+/m	3+/e

0: no cells in section are immunopositive (negative); 1+: < 10% of cells in section are immunopositive (minimal); 2+: 11–25% of cells in section are immunopositive (mild); 3+: 26–50% of cells in section are immunopositive (moderate); 4+: 50–75% of cells in section are immunopositive (marked); 5+: >75% of cells in section are positive (severe). e = endothelial cell, c = choroid ependymal cell, m = macrophage, h = hepatocyte, k = Kupffer cell, l = lymphocyte, p = pneumocyte.

### Ferret model of HTNV and PUUV infection

**Fig 5:**
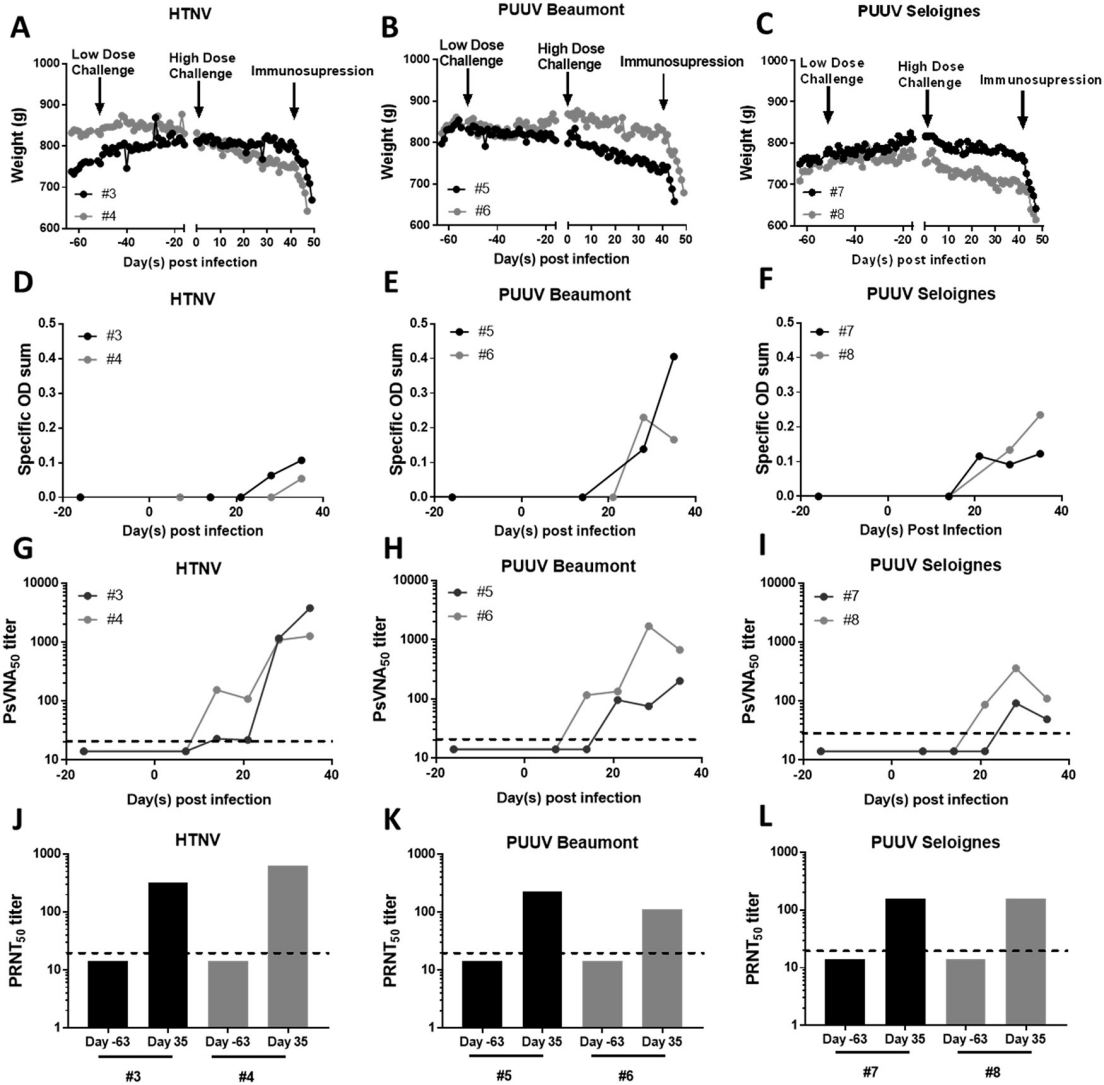
Ferrets are susceptible to HTNV and PUUV infection. Ferrets were challenged with either 2,000 PFU HTNV i.n., or 2,000 PFU PUUV i.n. (low dose challenge arrow) and did not seroconvert. The same animals were subsequently challenged with either 200,000 PFU HTNV (A,D,G,J), 94,000 PFU PUUV Beaumont (B,E,H,K), or 164,000 PFU of PUUV Seloignes (C,F,I,L) i.m. on day 0, and immunosuppressed with 30 mg/kg cyclophosphamide on day 41. The daily weight (g) for each ferret is displayed (A-C). Seroconversion of ferrets was monitored by N-ELISA specific OD sum (D-F), and neutralizing antibody production by PsVNA_50_ (G-I) and PRNT_50_ (J-L). The dashed line represents the limit of detection for the PRNT (20) and PsVNA (20) assay.

No published studies detail if ferrets are susceptible to hantavirus infection. To examine this, four ferrets were exposed to either 2,000 PFU HTNV or PUUV K27 i.n. No seroconversion occurred within 35 days post infection. The same animals were re-exposed to either 200,000 PFU HTNV, 94,000 PFU PUUV Beaumont (a human PUUV isolate) or 164,000 PFU PUUV Seloignes (a vole PUUV isolate) by i.m. Prior to the re-exposure one of the seronegative ferrets in the HTNV group was removed for health concerns (rapid weight loss) unrelated to the study, and it’s cage mate was subsequently removed for behavioral health reasons before the completion of the study. Data from those two ferrets are not shown. As soon as three days post infection ferrets began to lose weight with HTNV infected ferrets losing between 5–12% of peak body weight as did PUUV infected ferrets (Fig 5A–5C). By Day 35 post infection all animals had developed antibodies against all strains of the virus as measured by N-ELISA assay (Fig 5D–5F). Neutralizing antibody development began as early as Day 14 post infection and all ferrets developed neutralizing antibodies by Day 28 post infection (Fig 5G–5L). EDTA treated blood was analyzed weekly for evidence of leukocytosis and thrombocytopenia; none was observed (S6 Fig) nor was serum viremia detected by RT-PCR (S7A Fig).

Syrian hamsters infected with SNV do not develop lethal disease, unless immunosuppressed [6, 7]. On Day 42 post infection infected ferrets were administered 30 mg/kg Cyp. Within two days post administration the total white blood cell, lymphocyte and neutrophil count had decreased significantly (S8 Fig), and was almost zero seven days post administration. The immunosuppressed ferrets rapidly lost weight, became lethargic, with occasional vomiting and diarrhea. Animals were euthanized between 3 and 7 days post administration of Cyp having met humane endpoint criteria.

The organs of infected ferrets were analyzed for viral load by RT-PCR and for the presence of infectious virus by plaque assay. The lung, liver, spleen, intestine and urine of HTNV infected ferrets were negative for viral genome, with small amounts (<2 log_10_) detected in the heart and spleen of a single animal (S7B Fig). The heart, lung, kidney, intestine and urine of PUUV infected ferrets were negative for viral genome, though small amounts were detected in the liver (2/4) and spleen (1/4) of PUUV infected ferrets (S7C and S7D Fig). Viral genome was spiked into the assay to confirm that the lack of signal was not due to the presence of inhibitors. All organs except for the intestine (4/6) had no inhibition of RT-PCR product. Similarly, no infectious virus was found in the organ of any ferret by plaque assay, despite spiked-in virus exhibiting no significant inhibition (S9 Fig).

To confirm that immunosuppression of uninfected ferrets did not result in rapid weight loss, four healthy ferrets were immunosuppressed with a loading dose of 30 mg/kg Cyp followed by 10 mg/kg Cyp maintenance doses every other day (S10A Fig). Five days post immunosuppression ferrets exhibited rapid weight loss, fever, and lethargy due to secondary infection (S10B & S10C Fig). Immunosupression was discontinued and 5 mg/kg enrofloxicin (a broad spectrum antibiotic) was given twice daily for a week. During this time, ferrets began to gain weight and their fever diminished. On Day 11 immunosupression resumed for two doses with prophylactic enrofloxicin given once daily. Even with prophylactic antibiotics two ferrets spiked fevers within a few days post the second round of immunosuppressive treatment, though they did not lose weight (S10B & S10C Fig). Based on these results the rapid weight loss and clinical signs observed upon immunosuppression of HTNV and PUUV infected ferrets was most likely due to secondary infection. Due to the inability to completely manage secondary infection with prophylactic antibiotic treatment, no further immunosuppression studies were carried out in ferrets.

**Fig 6:**
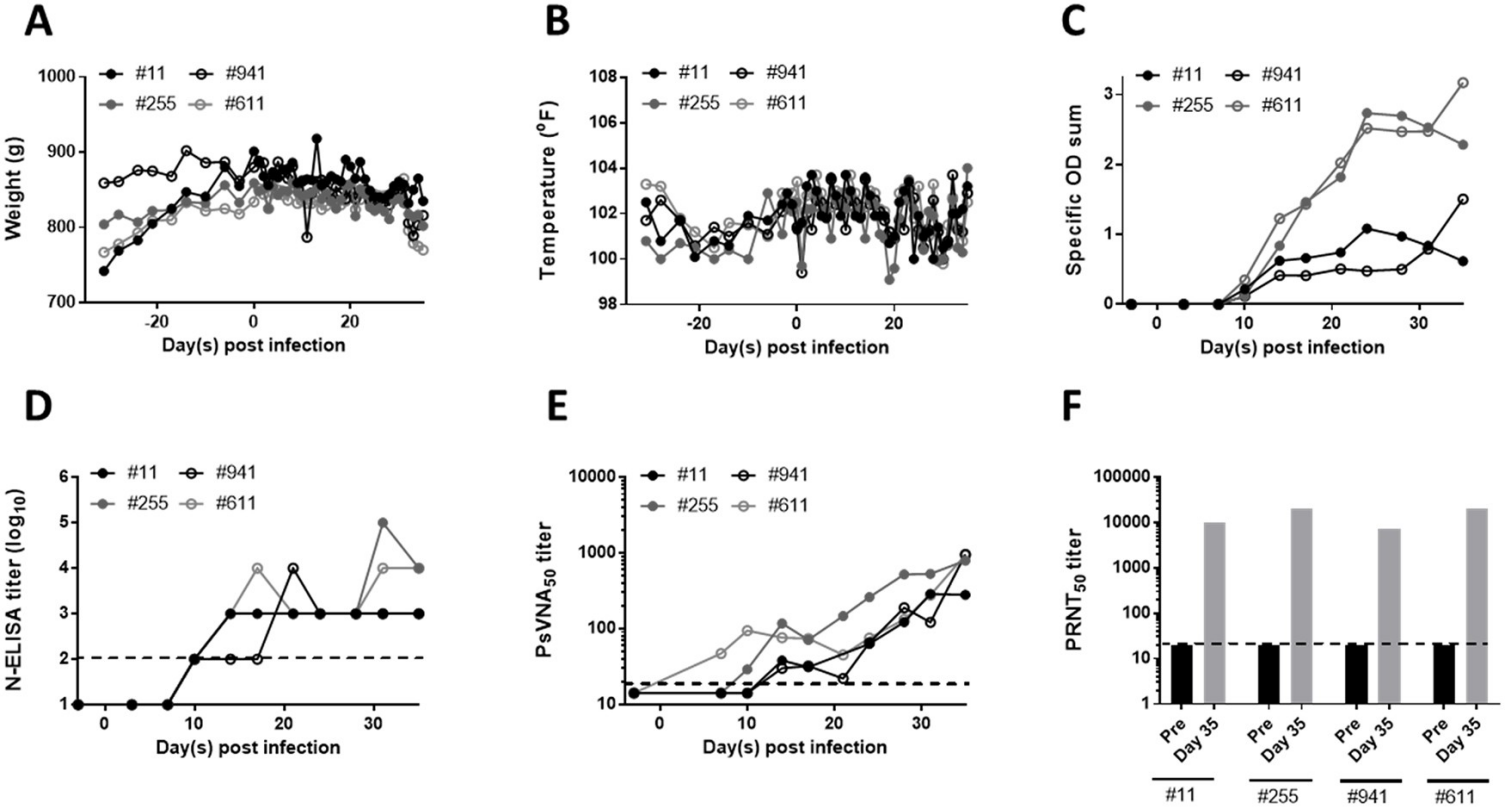
PUUV infected ferrets experience weight loss, but no other clinical signs of infection. Ferrets were infected with 94,000 PFU PUUV Beaumont i.m. and sera was collected twice weekly. Weight (A), temperature (B), N-ELISA specific OD sum (C), N-ELISA titer (log_10_), (D) PsVNA_50_ titers (E), and PRNT_50_ titers (F). The dotted line represents the limit of dectection for the PRNT assay (20), the PsVNA assay (20) and the N-ELISA (2log_10_).

To refine the HFRS-causing hantavirus ferret infection model four ferrets were challenged with 94,000 PFU PUUV Beaumont i.m. on Day 0. Weight and temperature were recorded daily, while twice weekly blood draws and urine collection was used to monitor kidney function. As with the pilot experiment, ferrets slowly lost between 7–11% of peak body weight, recapitulating our previous findings (Fig 6A). No elevated temperatures were observed (Fig 6B). Ferrets developed a robust antibody response beginning on Day 10 post infection (Fig 6C and 6D). Neutralizing antibodies developed early as Day 14 post infection, and were present in all ferrets by Day 28 post infection, as measured by PsVNA and PRNT (Fig 6E and 6F).

**Fig 7:**
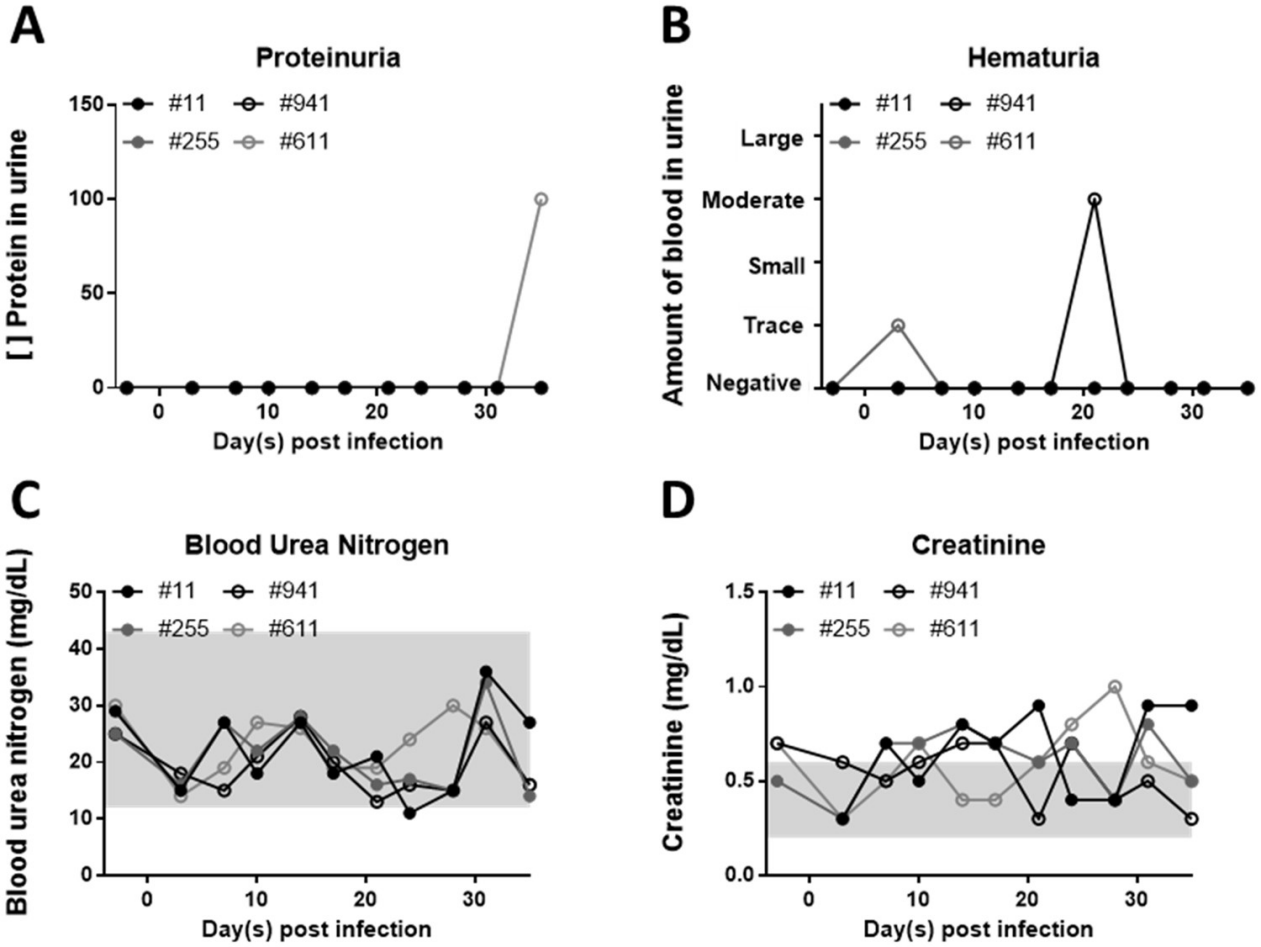
PUUV infected ferrets do not exhibit signs of kidney impairment. Ferrets were infected with 94,000 PFU PUUV Beaumont i.m. Blood and urine were collected twice weekly for analysis of kidney function by assaying proteinuria (A), hematuria (B), blood urea nitrogen (C), and creatinine (D) levels. Shaded gray areas represent the normal range [48].

Despite the slow weight loss no signs of renal impairment were observed. Proteinuria and hematuria are hallmarks of PUUV infection occurring in between 94–100% (proteinuria) and 58–85% (hematuria) of human clinical cases [46]. No prolonged proteinuria or hematuria was observed (Fig 7A and 7B) in infected ferrets. Similarly, blood urea nitrogen and creatinine levels in the sera, both of which are elevated due to kidney failure in PUUV patients [47] remained unchanged in PUUV infected ferrets (Fig 7C and 7D) [48, 49]. No infectious virus or viral genome was detected in the brain, heart, lung, liver, spleen, kidney, intestine, or eye (S11 and S12 Figs). No changes in other serologic or urologic parameters were noted (S13 and S14 Figs).

No gross pathological changes or significant lesions associated with PUUV infection were noted in the ferrets (S15 Fig). In the lungs, one ferret had mild neutrophilic and histiocytic inflammation centered on the bronchioles and expanded alveolar septa. Given that the inflammation was centered around bronchioles and not the vasculature, it is unlikely to be in response to PUUV infection. A number of other common or age-associated lesions were observed in the ferrets. Two of four ferrets had proliferative cortical cells in either the adrenal capsule or adrenal cortex that were likely clinically silent as they lack clinical signs consistent with adrenal–associated endocrinopathy. Additionally, alveolar mineralization was noted in all four ferrets as was eosinophilic and lymphoplasmacytic enteritis, and hepatitis. Two ferrets had fibromyxomatous degeneration of the atrioventricular valve. The spleen, brain, kidney, and pituitary gland were normal in all ferrets examined.

### Marmoset model of HTNV infection

**Fig 8:**
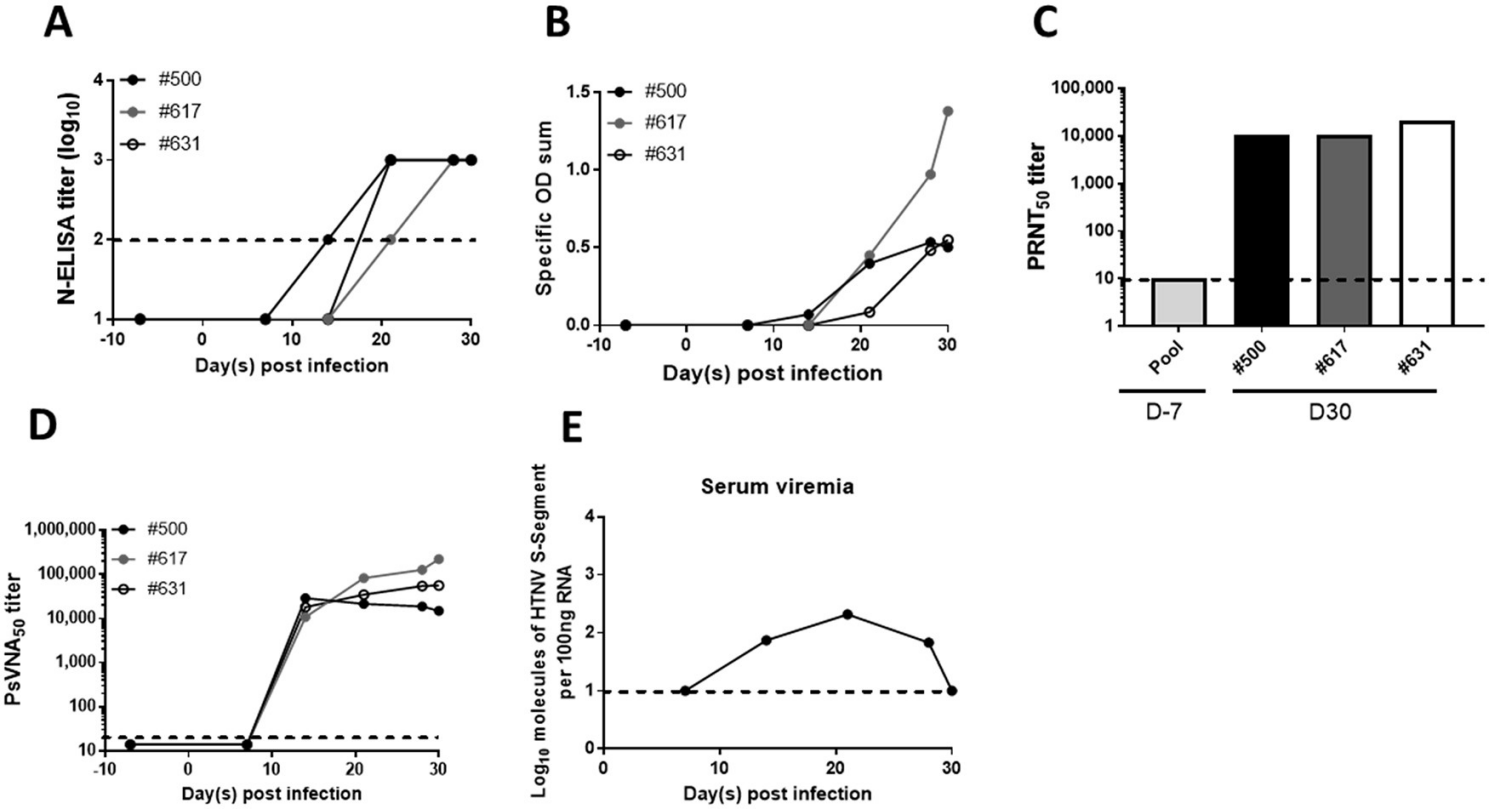
Marmosets are susceptible to HTNV infection. Three marmosets were infected with 1,000 PFU HTNV i.m. Sera was collected weekly to monitor seroconversion. N-ELISA titer (log_10_) (**A**) N-ELISA specific OD sum (**B**) PRNT_50_ (**C**) and PsVNA_50_ titers(**D**) are displayed. Serum viremia was assayed by RT-PCR (**E**). The limit of detection for N-ELISA titer (2log_10_), PRNT (20), PsVNA (20) and RT-PCR (1.0log_10_) is depicted by the dotted line.

As with ferrets, there is no literature on the susceptibility of marmosets to hantavirus infection. To test this, three male marmosets were exposed to 1,000 PFU HTNV i.m. Blood was collected weekly to measure seroconversion, serum viremia, as well as serum parameters relating to renal function. All three animals seroconverted by Day 21 post infection (Fig 8A and 8B) despite displaying no clinical signs of illness. Neutralizing antibody production began around the same time, and was robust by Day 30 post infection (14,866–221,557 by PsVNA_50_ and 10,240–20,480 by PRNT_50_) (Fig 8C and 8D). Due to low volumes of blood drawn at each time point serum viremia could not be examined for each individual animal, however a pool of sera from all three animals was evaluated at each time point post infection. Low levels of serum viremia were detected between days 14 and 28 post infection by RT-PCR (Fig 8G). As with the ferret infection model of HFRS-causing hantavirus, no renal injury as measured by changes noted in blood urea nitrogen or serum creatinine were observed over the course of infection, nor were changes in any of the other serum parameters monitored (S16 Fig).

Animals were euthanized on Day 30 post infection to examine organs for viral dissemination. No infectious virus or viral genome was detected in the heart, lung, liver, spleen, kidney, intestine or brain (S17 and S18 Figs). Lymphoid hyperplasia was noted in all three animals by histology, though the location and intensity varied between the spleen, various lymph nodes and gut associated lymphoid tissue. Such hyperplasia is indicative of a response to antigenic stimulation and was most likely caused by the viral challenge. Mild to moderate congestion was also seen in the lungs of each marmoset; however, this was an acute change and was most likely associated with terminal anesthesia and euthanasia. No other significant histological lesions were noted in any of the three animals (S19 Fig). Due to the negative virology results and the lack of significant histologic changes, IHC to detect the presence of HTNV antigen in tissues was not performed.

## Discussion

To date, the use of adult animal models to evaluate anti-HTNV and anti-PUUV medical counter measures has been limited. Recombinant protein, vaccinia virus-vectored, and DNA vaccines have been tested in the high dose HTNV hamster model [9, 37, 50]. Additionally, the ability of passively administered neutralizing antibodies has been evaluated in the high-dose hamster model of HTNV and PUUV and in PUUV challenged cynomolgus macaques PUUV [50, 51]. Both of these models have limitations; the size of the macaque requires large quantities of passive transfer material, and the high dose of the hamster model, with challenge doses of ~650 ID_99_, could require prohibitively large volumes of test article to neutralize the high initial dose. Suckling mice, which present with a disseminated disease not reminiscent of HFRS, have been used to evaluate HTNV therapeutics as well [52–55]. In this paper we present three adult animal models of HFRS-causing hantavirus infection than can be used for future evaluation of therapeutics, biologics and vaccines.

### Low-dose hamster model of HFRS-hantavirus infection

The ID_50_’s for HTNV and PUUV determined in this report are similar to the lethal dose 50% (LD_50_) calculated for SNV and ANDV, <3 PFU via the i.m. route [6, 7, 41]. Also similar is that the challenge dose of ANDV required to infect/kill 50% of hamsters by the i.n. or intragastric route is ~10–30 fold higher [56]. Previous reports also demonstrate that PUUV is capable of infecting hamsters by the intragastric route, with an ID_50_ of >10,000, making it a much less effective route of infection [57]. Despite similarities in their ID_50_’s, the ID_99_’s of HTNV and PUUV greatly diverge, with ~200 times less HTNV required to infect hamsters via the i.m. route, and ~2 fold less required to infect via the i.n. route (Table 1). The lower ID_99_ doses, coupled with viral persistence in HTNV infected animals as opposed to viral clearance (Fig 3), suggest that HTNV is more infectious than PUUV in the hamster. The mechanism for this difference between these closely related hantaviruses remains unknown.

Lethal infection of hamsters with ANDV leads to extensive organ dissemination, with infectious virus recovered in the lung, liver, kidney, spleen and heart [6]. Asymptomatic infection of hamsters with SNV repeatedly passaged through hamsters has a similar organ distribution [58]. The organ distribution of both those viruses is similar to the low dose HTNV hamster model, with two notable exceptions. First, the HTNV model has low, transient levels of virus in the liver and hardly any virus in the spleen (Figs 3 and 4). This dissimilarity between the models led us to confirm the lack of virus in the spleen was not due to the presence of inhibitors by spiking either infectious virus or viral RNA into samples prior to evaluation (S2 Fig). Second, in the ANDV model, the presence of virus was determined by plaque assay, indicating the virus was infectious and replication competent. In the HTNV low dose hamster model, while there is detection of high levels of viral genome (and in the case of the HTNV i.m. model, by pathology) by RT-PCR, recovery of infectious virus is sporadic, typically occurring at low levels, in only a few hamsters per time point (Figs 3 and 4, Table 1). The discrepancy between RT-PCR/pathology and plaque assay is notable. The hamsters in this study were not perfused, and given the appearance of neutralizing antibodies as early as day XX post infection, the presence of these antibodies could be impairing out ability to recover live virus via the plaque assay. It is also possible that viral packaging is somehow impaired in the hamster, leading to a larger amounts of nucleocapsid protein and viral genome than live virus. Further studies need to be undertaken to elucidate.

The low dose HTNV hamster model also mimics the infection pattern of the virus in its host species, the striped field mouse (*Apodemus agrarius)*, with viral genome being detected in the lung, liver and kidney but not the spleen (the heart and brain were not examined) [32, 59–61].

In contrast to the HTNV low dose model, the organ distribution of virus in the PUUV low dose model is transient. In PUUV i.m. infected hamsters virus is detectable by RT-PCR around Day 11 post infection, with the virus being cleared from all organs except the lung by Day 24. No infectious virus was recovered at any time point examined. Even less virus was detected in the PUUV i.n. model; only a small amount in the brain of one hamster on Day 28 post infection. In neither case was serum viremia observed (Figs 3 and 4). This is most similar to the SNV models involving low passage virus in immunocompetent hamsters: the virus is transiently detected in the lung by PCR, and then sporadically found in organs 12 to 14 days post infection using immunohistochemistry [41, 58]. The distribution of PUUV in the hamster differs somewhat from its host species the bank vole, where it is found to persist in the lung, spleen and kidney, and was not detected in the heart or the brain [62].

Seroconversion of hamsters post viral exposure remains the best way to measure infection, and should be a considered the primary endpoint for efficacy studies. Though the PRNT assay was slightly more sensitive than the N-ELISA assay, detecting neutralizing antibodies in all animals with N-ELISA titers, and in two PUUV animals that did not have N-ELISA titers, the increased time, sample, and biosafety requirements necessary for a PRNT assay make the N-ELISA a better choice (Fig 2, S1 Fig). For a 10 PFU HTNV i.m. challenge, given lower titer and specific OD sum values as compared with higher challenge doses, and the fact that one hamster with significant viral genome in its organs at Day 28 post infection (7.1 log_10_ in the brain, and 6.8 log_10_ in the kidney) did not seroconvert, waiting until Day 35 post infection to monitor seroconversion is advisable (Figs 1–3). Viral load in the brain, kidney, and lungs as measured by RT-PCR need to be evaluated at Day 35 post low-dose challenge to determine their usefulness as secondary endpoints. Recovery of infectious virus in any organ, and viremia are too sporadic to serve as proxy markers for infection.

### Ferret infection model

The ferret has been used as an experimental model for numerous hemorrhagic fever viruses, and respiratory viruses [12–17], though no published reports exist examining its susceptibility to hantavirus infection. In comparison to the hamster, the ferret is far more resistant to HTNV and PUUV infection. Exposure of ferrets to 2,000 PFU i.n. (greater than the ID_99_ for both viruses in hamsters), failed to result in a productive infection and seroconversion. Instead i.m. challenge doses of ~100,000–200,000 PFU were needed (Fig 5). Initially ferrets were exposed to PUUV K27, a commonly used laboratory isolate that has been in cell culture for over 15 years. Repeated passaging of hantavirus is known to cause mutations [63–65]. In contrast PUUV Beaumont and Seloignes are relatively recent isolates, with no more than 3 passages in cell culture post isolation. These strains were used for all subsequent experiments to maximize the likelihood of PUUV to cause disease by eliminating possible attenuation of the laboratory strain of the virus. Despite the high challenge dose and use of recent isolates, no elevation in white blood cells was observed over the course of the experiment, no pathology or organ burden was detected at the conclusion of the experiment, and the N-ELISA specific OD sum, PRNT_50_, and PsVNA_50_ titers remained low (Figs 6 and 8 and S9 Fig). This outcome is almost identical to that of Marburg and Ravn virus infection in ferrets, where the development of neutralizing antibody titers was the only sign that productive infection occurred [66]. Previous experiments in hamsters, have demonstrated that 2x10^5^ PFU of gamma-irradiated ANDV and SNV are not sufficient to cause seroconversion, and neither is 1x10^4^ gamma-irradiated PFU PUUV [6, 57]. Thus the seroconversion observed in ferrets, though low, is not likely to be due to a reaction to the large quantity of antigen administered, but to a productive infection.

PUUV antigen, viral genome, or infectious virus has been found in the brain, pituitary gland, lung, heart, liver, kidney, spleen, cerebrospinal fluid, and gastrointestinal tract of human patients with clinical symptoms of NE, though the pattern of viral dissemination varies between individuals [67–70]. Acute kidney injury and vision disturbances including blurred vision, myopic shift, and lens thickening, while pulmonary involvement including pleural effusion and vascular congestion, and renal failure occurred less frequently [47, 71–74]. Given the lack of high neutralizing antibody titers, which could have aided in viral clearance, the lack of viral genome in any of the ferret organs examined is rather surprising (S6, S7, S11 and S12 Figs). Furthermore, the lack of viral antigen and pathology in the organs tested suggest either a transient infection cleared prior to Day 35 post infection, levels of virus so low as to be undetectable by the tests used, or a viral reservoir outside of the organs tested.

The lack of detectable virus is most surprising given the gradual weight loss infected animals exhibit (Figs 5 and 6). The animals gained weight until ~3 days post challenge, at which point a gradual weight loss occurs, regardless of if HTNV or PUUV was the challenge virus. While weight loss is a feature of other ferret models of infectious diseases, the pattern we observed was unique: ferrets infected with morbillivirus, avian influenza and filoviruses rapidly lose weight during the first week to two weeks post infection, while infection with severe acute respiratory syndrome virus results in no significant weight loss [16, 17, 75–77].

In the hamster, infection with SNV is asymptomatic unless the hamster is immunosuppressed. When ferrets were immunosuppressed on Day 42 post infection, rapid weight loss and lethargy ensured (Fig 5). Given that these clinical signs were also observed in unchallenged control animals (S10 Fig), this could likely be the result of a secondary infection or drug toxicity. The use of Cyp is well documented in ferrets, primarily given at a high dose as an emetic, and no dosage for long term immunosuppression was found [78]. The dosages used in this study (between 10–30 mg/kg) successfully reduced white blood cell levels, in ferrets and demonstrate that Cyp can be used to induce long-term immunosuppression, if antibiotics are given to control for secondary infection (S8 Fig).

Despite not being able to detect infectious virus or viral genome in the kidney, we hypothesized that the weight loss we observed could be due to kidney failure. Individuals with HFRS exhibit proteinuria and hematuria, both of which can indicate kidney damage [46, 47]. Additionally, serum blood urea nitrogen and creatinine levels are both elevated in HFRS patients and provide a second way to measure kidney function [46, 47, 73]. Decreased platelet levels also characterize clinical HFRS in humans, impairing coagulation [46, 47, 73]. In a second experiment designed to monitor kidney parameters, PUUV infected ferrets exhibited the same gradual weight loss that characterized the first experiment. However, no prolonged signs of clinical kidney failure were observed: blood urea nitrogen and creatinine levels did not dramatically increase over the five week study period. Only one animal exhibited proteinuria (day 35), and two exhibited hematuria (one on Day 4, and one on Day 21) (Fig 7). Similarly to parameters monitoring renal failure, no thrombocytopenia occurred in PUUV infected ferrets (S9 and S13 Figs). The cause of the weight loss remains undetermined.

Though susceptible to both HTNV and PUUV, the ferret has limited usefulness for studies involving medical countermeasure efficacy testing. The ferret’s large mass, even as an adolescent, makes the amount of test article needed also prohibitively large. The large challenge dose required for infection could potentially obscure the protective effect of drugs of vaccines, due to the overwhelming amount of virus administered. Moreover, although the animals are infected the resultant neutralizing antibody titers are small, resulting in potential sensitivity issues with the model. Husbandry and handling of the animals under ABSL-3 procedures is also substantially more difficult than hamsters, and they lack the genetic similarity to humans that marmosets possess.

### Marmoset infection model

In this study we have demonstrated the susceptibility of marmosets to HTNV infection. Marmosets represent an attractive model for testing vaccines and therapeutics against HFRS-causing hantaviruses due to genetic similarities to humans and small size. Also, the model has a simple read-out of infection, i.e. robust antibody production as measured by N-ELISA and PsVNA, making the determination of protection by vaccine or passive transfer material, straightforward. Further optimization of the model, namely to determine the ID_99_, could prove important as a 1,000 PFU challenge dose could be excessively high and prohibit therapeutic effects of candidate medical countermeasures.

Overall the marmoset model is more similar to the ferret HFRS-causing hantavirus infection model than the hamster, though there are key differences. Like the ferret, no significant pathological abnormalities were noted, and no signs of renal failure were observed (S16 and S19 Figs). Serum chemistry values do not differ from the normal range with the exception of albumin, total bilirubin, and amylase. While these values fell outside the normal range, they did not change over the course of infection indicating the problem may lie in the reference values used. The Piccolo general chemistry panel used to evaluate the parameters is optimized for human testing, and therefore may be less than optimal for evaluating the marmoset, especially those parameters. Additionally, no infectious virus or viral genome was recovered from any organ at Day 30 post infection [79, 80]. This is not surprising, given the high levels of neutralizing antibodies present as early as 21 days post infection (Fig 8). Unlike the ferret, however, marmosets develop exceptionally high neutralizing antibody titers (10,240–20,480 by PRNT_50_ and 14,866–221,557 by PsVNA_50_), and display low-level serum viremia between two and four weeks post infection (Fig 8). The serum viremia is significantly lower than in hamsters infected with HTNV, where some animals displayed RT-PCR titers of >7log_10_, and in hamsters infected with ANDV, where infectious virus titers prior to death are > 6log_10_ [41].

Despite not exhibiting clinical signs of disease, the model’s robust antibody response (as measured by PRNT, PsVNA and N-ELISA) make it a useful tool for evaluating vaccines and pre-or post-exposure therapeutics.

### Conclusion

This paper has explored the use of three laboratory animal species as possible infection and disease models for HFRS-causing hantaviruses: the hamster, the ferret, and the marmoset. These models, especially the hamster model and marmoset model, will be useful for evaluating medical countermeasures with the potential to induce sterile immunity. The marmosets should be particularly useful for the evaluation of passively transferred protective human antibodies because of the relative genetic similarities between species in the Order Primates, and the small size of marmosets, allowing testing with smaller volumes of material than would be required for larger species such as macaques.

## Supporting information

S1 Fig Seroconversion in low dose hamster models. Syrian hamsters were infected either 10 PFU HTNV i.m. (**A**), 500 PFU HTNV i.n. (**B**), 1000 PFU PUUV i.m. (**C**), or 1000 PFU PUUV i.n. (**D**). No sera was available for one hamster in the Day 28 HTNV i.m. group. Hamsters sacrificed on days 17–28 had their sera screened for neutralizing activity by PRNT. The mean titer is displayed for each group, and the limit of detection for the assay (20) is depicted by a dashed line.
(TIF)

S2 Fig Lack of viral RNA in the spleen of HTNV infected animals is not due to inhibitors. Syrian hamsters were infected either 10 PFU HTNV i.m. or 500 PFU HTNV i.n. Hamsters were terminally bled at various points post infection. Samples were evaluated with and without the addition of exogenous viral genome (by RT-PCR) (**A&B**) and randomly selected negative samples were evaluated with and without infectious virus by at the 1:10 dilution by plaque assay (**C&D**). The mean ± the SEM is shown for each group and the limit of detection for each (RT-PCR LOD = 1 log_10_; Plaque assay = 50 (1.7 log_10_) plaques) is displayed as a dashed line.
(TIF)

S3 Fig Repeated cell culture passaging can result in detection of infectious virus in the urine. Syrian hamsters were infected either 10 PFU HTNV i.m. or 500 PFU HTNV i.n. Hamsters were euthanized and urine was collected at various points post infection. Three RT-PCR positive, and three RT-PCR negative urine samples underwent amplification by cell culture. (**A**) Schematic of urine amplification strategy. Red arrow indicates infection of Vero E6 cells, purple arrow indicates sample collection. On Day 4 supernatant was collected and frozen, and used to infect fresh Vero E6 cells at a later date. (**B**) Presence of viral genome over the course of amplification as tested by RT-PCR. (**C**) Pre- and post-amplification plaque assay results with the mean titer is displayed for each group as a solid line. The limit of detection for each (RT-PCR LOD = 1 log_10_; Plaque assay = 1.1 log_10_) is displayed as a dashed line. (POS) is virus spiked into water (**B**) or media (**D**) to serve as a positive control.
(TIF)

S4 Fig HTNV and PUUV infection do not lead to changes in hematological parameters. Syrian hamsters were infected either 10 PFU HTNV i.m., 500 PFU HTNV i.n., 1000 PFU PUUV i.m., or 1000 PFU PUUV i.n. Whole blood was collected at the time of euthanasia and evaluated for white blood cell count (**A**), red blood cell count (**B**), hematocrit (**C**), platelets (**D**), neutrophils (**E**), lymphocytes (**F**), monocytes (**G**), eosinophils (**H**), basophils (**I**). The gray box indicates the normal range of hamsters as determined by uninfected control animals.
(TIF)

S5 Fig Viral dissemination to organs in HTNV i.m. infected hamsters as detected by IHC. Hamsters were infected with 10 PFU HTNV i.m and sacrificed at various time points post infection. Heart, lung, liver, spleen, kidney and brain tissue were fixed in formalin, sectioned, and stained by IHC to identify HTNV viral antigen. Representative images of organs from normal and Day 28 are shown. Pictures at 400x magnification.
(TIF)

S6 Fig HTNV or PUUV infection does not cause any changes in white blood cell levels. Ferrets were challenged with either 200,000 PFU HTNV, 94,000 PFU PUUV Beaumont, or 164,000 PFU of PUUV Seloignes i.m. Whole blood was drawn weekly post infection and evaluated for White blood cell count (A), platelets (B), neutrophils (C), lymphocytes (D), monocytes (E), eosinophils (F), basophils (G). The gray box represents the average range of values for ferrets [81].
(TIF)

S7 Fig No appreciable viral genome was detected in HTNV and PUUV infected ferrets. Ferrets were challenged with either 200,000 PFU HTNV, 94,000 PFU PUUV Beaumont, or 164,000 PFU of PUUV Seloignes i.m. on Day 0, and immunosuppressed with 30 mg/kg Cyp on Day 41. Sera was collected weekly and assayed for serum viremia by RT- PCR (**A**). At time of euthanasia, heart, lung, liver, spleen, kidney, intestine and urine (except #7) were assayed by RT-PCR for the presence of viral genome. No appreciable genome was recovered in ferrets infected with HTNV (**B**), PUUV Beaumont (**C**) or PUUV Seloignes (**D**). Virus was spiked into the samples to confirm no inhibitor was present. The limit of detection for RT-PCR is 1 log_10_ and is represented by the dashed line. (POS) is virus spiked into water to serve as a control.
(TIF)

S8 Fig Immunosuppression with Cyp decreases ferret white blood cell counts. Ferrets were challenged with either 200,000 PFU HTNV, 94,000 PFU PUUV Beaumont, or 164,000 PFU of PUUV Seloignes i.m. and immunosuppressed with 30 mg/kg Cyp every other day beginning on Day 41. Whole blood was drawn from ferrets to evaluate white blood count (WBC) (**A**), lymphocyte count (**B**) and neutrophil count (**C**) prior to, two days post, and seven days post Cyp administration (if alive). Line depicting mean is shown. Student t-test or Mann–Whitney test was used to compare values between pre-Cyp and Cyp day 2 depending on the standard deviation of the groups being compared. As only two ferrets survived until Cyp Day 7 no statistics are possible. **** P<0.0001.
(TIF)

S9 Fig No infectious virus was recovered from the organs of HTNV or PUUV infected ferrets. Ferrets were challenged with either 200,000 PFU HTNV, 94,000 PFU PUUV Beaumont, or 164,000 PFU of PUUV Seloignes i.m. on Day 0, and immunosuppressed with 30 mg/kg Cyp on Day 41. Heart, lung, liver, spleen, kidney, and intestine were collected and assayed by plaque assay for the presence of infectious virus (**A**). To confirm lack of virus recovered was not due to inhibitors, virus was spiked into serial dilutions of organ homogenate to confirm no inhibitor was present (**B**). For a standard plaque assay the limit of detection, 1.7 log_10_, is depicted as a dashed line in (**A**). In (**B**) the dashed line is amount of HTNV plaques obtained when spiked into media rather than organ homogenate.
(TIF)

S10 Fig Immunosuppression of uninfected ferrets leads to rapid weight loss and secondary bacterial infection. Uninfected ferrets were administered 10mg/kg Cyp, and the antibiotic enrofloxicin, according to the schedule in (**A**). Weight (**B**) and temperature (**C**) are shown. b.i.d indicates antibody was administered twice daily, and q.d indicates antibiotic was administered daily.
(TIF)

S11 Fig PUUV infected ferrets had no infectious virus in the organs. Ferrets were infected with 94,000 PFU PUUV Beaumont i.m. Heart, lung, liver, spleen, kidney, intestine, brain, eye, and adrenal gland were collected on day 35 post infection and assayed for infectious virus by plaque assay (**A**). Virus was spiked in to confirm no inhi bitor was present (**B**). For a standard plaque assay the limit of detection, 1.7 log_10_, is depicted as a dashed line in (**A**). In (**B**) the mean ± SEM is depicted in all spiked groups and the dashed line is amount of HTNV plaques obtained when spiked into media rather than organ homogenate.
(TIF)

S12 Fig PUUV infected ferrets had no viral genome in their organs. Ferrets were infected with 94,000 PFU PUUV Beaumont i.m. Heart (**A**), lung (**B**), liver (**C**), spleen (**D**), kidney (**E**), intestine (**F**), brain (**G**), and eye (**H**), were collected on Day 35 post infection and assayed for viral genome by RT-PCR. Viral genome was spiked in to confirm no inhibitor was present. The mean ± SEM is depicted and the limit of detection, 1 log_10_, is shown as a dashed line. (NS) not spiked, (S) spiked and (POS) virus spiked into water.
(TIF)

S13 Fig No blood chemistry changes occurred in ferrets infected with PUUV. Ferrets were infected with 94,000 PFU PUUV Beaumont i.m. Sera was collected twice weekly for blood chemistry analysis. Sodium (**A**), potassium (**B**), total CO_2_ (**C**), chlorine (**D**), calcium (**E**), glucose (**F**), albumin (**G**), total protein (**H**), ALP (**I**), ALT (**J**), AST (**K**), total bilirubin (**L**). Shaded gray areas represent normal range (all reference values except tC0_2_ from [48], tC0_2_ from [49]).
(TIF)

S14 Fig Urinalysis remained normal after PUUV infection of ferrets. Ferrets were infected with 94,000 PFU PUUV Beaumont i.m. Urine was collected twice weekly for urinalysis. Leukocytes (**A**), nitrite (**B**), urobilinogen (**C**), pH (**D**), specific gravity (**E**), ketone (**F**), bilirubin (**G**), and glucose (**H**).
(TIF)

S15 Fig Organs from PUUV infected ferrets do not display virus-associated pathology. Ferrets were euthanized 35 days post a 94,000 PUUV Beaumont i.m. challenge. Heart, lung, liver, kidney, spleen, small intestine, adrenal gland, pituitary gland, cerebellum and cerebrum were evaluated by H&E. Pictures taken at 10x magnification. ND, no data.
(TIF)

S16 Fig No changes in HTNV infected marmoset blood chemistry occurred. Three marmosets were infected with 1,000 PFU HTNV i.m. Sera was collected weekly for blood chemistry analysis. Glucose (**A**), GGT (**B**), amylase (**C**), calcium (**D**), ALT (**E**), AST (**F**), ALP (**G**), total bilirubin (**H**), total protein (**I**) albumin (**J**), blood urea nitrogen (**K**), and creatinine (**L**) were measured. Gray shaded area represents reference values (all reference values except GGT and bilirubin from [79], reference values for GGT and bilirubin from [80]).
(TIF)

S17 Fig Marmoset do not have infectious virus in their organs after HTNV infection. Three marmosets were infected with 1,000 PFU HTNV i.m. On Day 30 post infection organs were harvested, and the presence of infectious virus was determined by plaque assay (**A**). To confirm no inhibitors were present, virus was spiked into samples (**B**). For a standard plaque assay the limit of detection, 1.7 log_10_, is depicted as a dashed line in (**A**). In (**B**) the mean ± SEM is displayed for all spiked groups, and the dashed line is amount of HTNV plaques obtained when spiked into media rather than organ homogenate.
(TIF)

S18 Fig Marmoset do not have viral genome in their organs after HTNV infection. Three marmosets were infected with 1,000 PFU HTNV i.m. On Day 30 organs were harvested, and the presence of viral genome was determined by RT-PCR. To confirm no inhibitors were present, viral genome was spiked into samples. Heart (**A**), lung (**B**), liver (**C**), spleen (**D**), kidney (**E**), intestine (**F**), and brain (**G**) were collected. The mean ± SEM is shown for the not spiked (NS) and spiked (S) groups and the limit of detection, 1 log_10_, is depicted as a dashed line. (POS) is viral genome spiked into water.
(TIF)

S19 Fig Organs collected from HTNV infected marmosets do not display virus-associated pathology. Marmosets were euthanized 30 days post 1,000 PFU HTNV i.m. challenge. Heart, lung, liver, kidney, spleen, small intestine, adrenal gland, pituitary gland, cerebrum, and cerebellum from normal and infected animals were stained by H&E for gross pathological changes. Representative images in this Fig are taken from all three animals. Pictures at 10x magnification.
(TIF)
