# Supplementary figures and images for: Three asymptomatic animal infection models of hemorrhagic fever with renal syndrome caused by hantaviruses
Source: PLoS One. 2019 May 10;14(5):e0216700. doi: 10.1371/journal.pone.0216700 (PMC6510444; doi:10.1371/journal.pone.0216700)

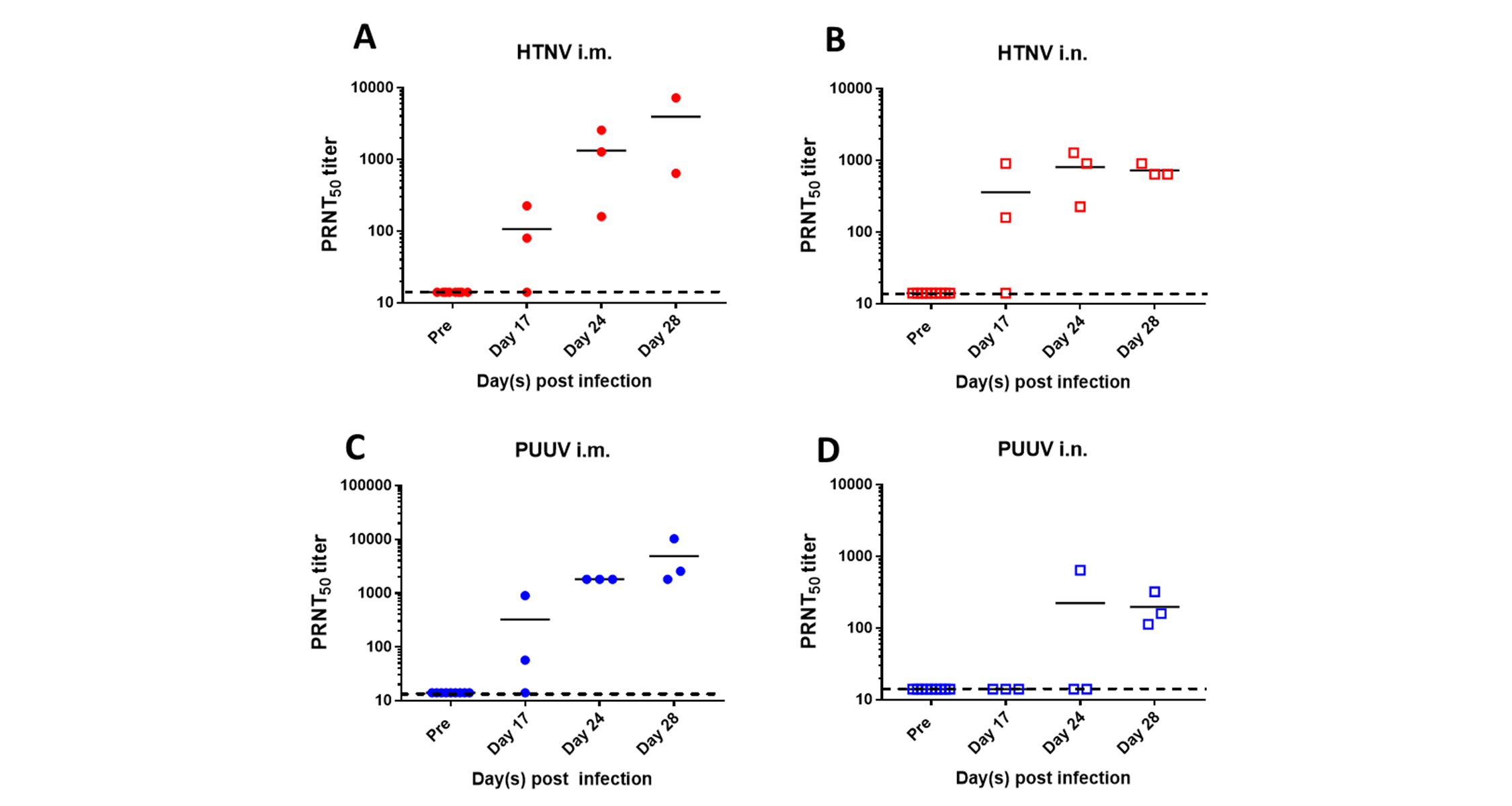

Supplement: S1 Fig — Syrian hamsters were infected either 10 PFU HTNV i.m. (A), 500 PFU HTNV i.n. (B), 1000 PFU PUUV i.m. (C), or 1000 PFU PUUV i.n. (D). No sera was available for one hamster in the Day 28 HTNV i.m. group. Hamsters sacrificed on days 17–28 had their sera screened for neutralizing activity by PRNT. The mean titer is displayed for each group, and the limit of detection for the assay (20) is depicted by a dashed line. (TIF) [file pone.0216700.s001.tif]

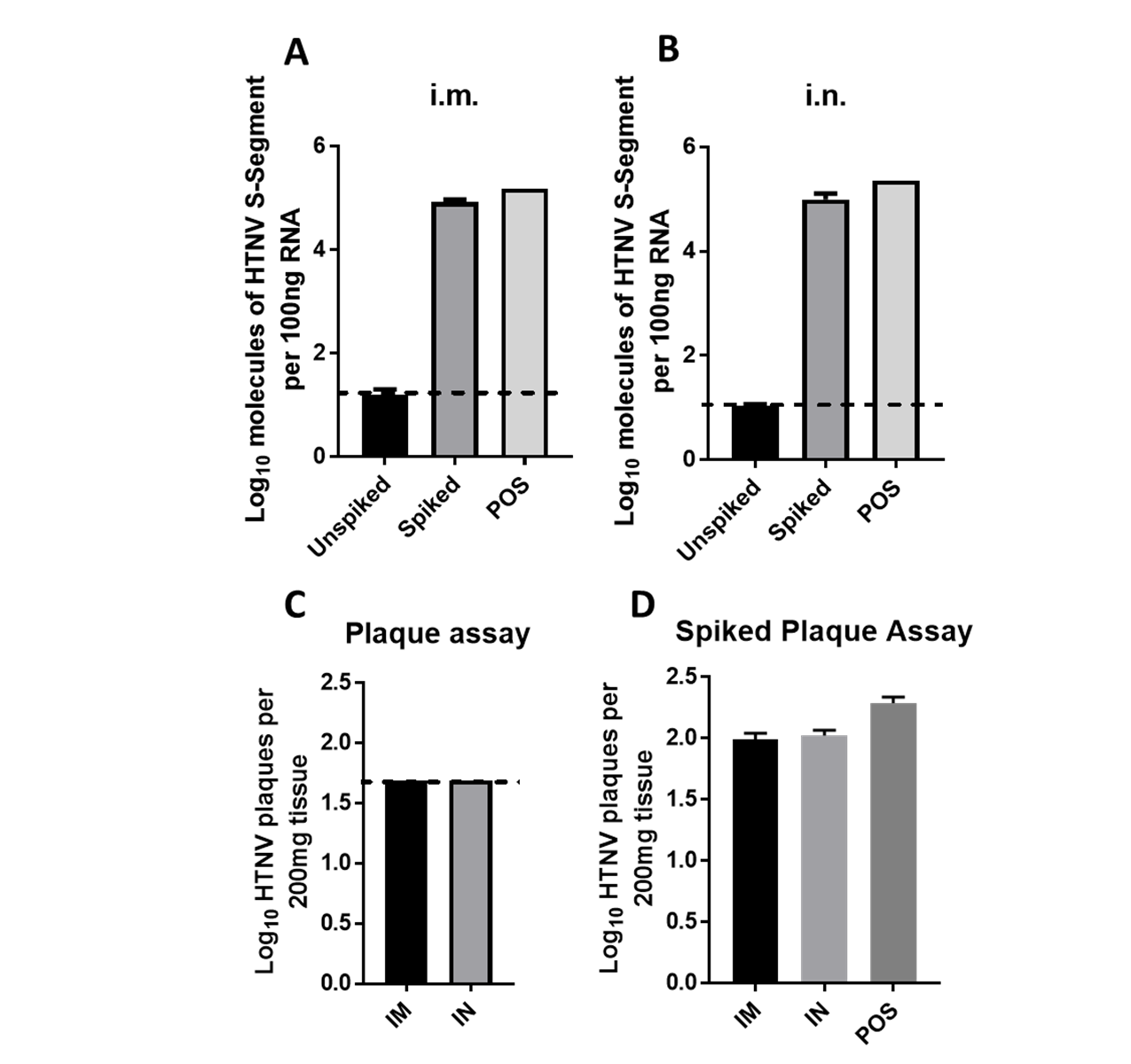

Supplement: S2 Fig — Syrian hamsters were infected either 10 PFU HTNV i.m. or 500 PFU HTNV i.n. Hamsters were terminally bled at various points post infection. Samples were evaluated with and without the addition of exogenous viral genome (by RT-PCR) (A&B) and randomly selected negative samples were evaluated with and without infectious virus by at the 1:10 dilution by plaque assay (C&D). The mean ± the SEM is shown for each group and the limit of detection for each (RT-PCR LOD = 1 log10; Plaque assay = 50 (1.7 log10) plaques) is displayed as a dashed line. (TIF) [file pone.0216700.s002.tif]

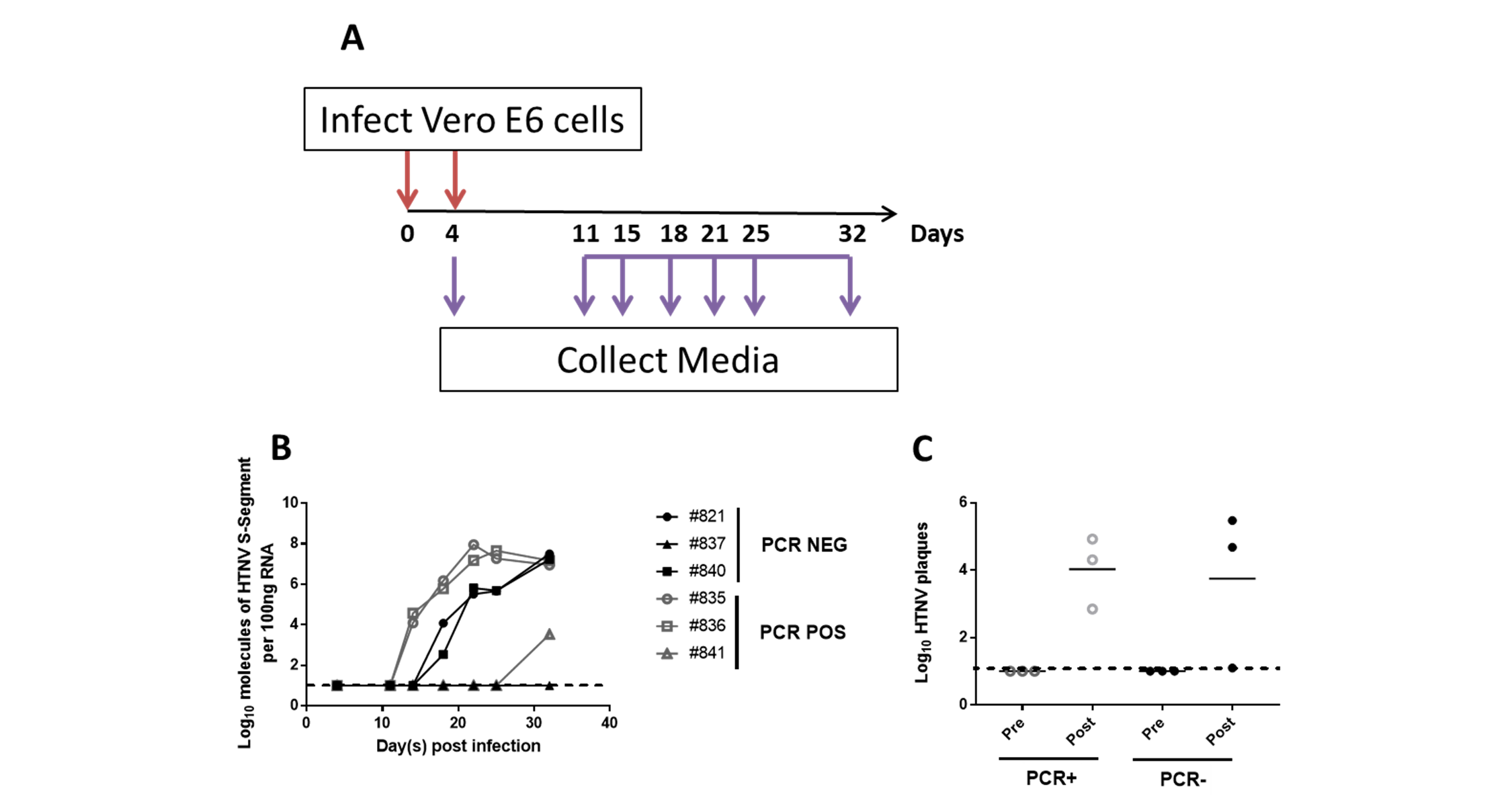

Supplement: S3 Fig — Syrian hamsters were infected either 10 PFU HTNV i.m. or 500 PFU HTNV i.n. Hamsters were euthanized and urine was collected at various points post infection. Three RT-PCR positive, and three RT-PCR negative urine samples underwent amplification by cell culture. (A) Schematic of urine amplification strategy. Red arrow indicates infection of Vero E6 cells, purple arrow indicates sample collection. On Day 4 supernatant was collected and frozen, and used to infect fresh Vero E6 cells at a later date. (B) Presence of viral genome over the course of amplification as tested by RT-PCR. (C) Pre- and post-amplification plaque assay results with the mean titer is displayed for each group as a solid line. The limit of detection for each (RT-PCR LOD = 1 log10; Plaque assay = 1.1 log10) is displayed as a dashed line. (POS) is virus spiked into water (B) or media (D) to serve as a positive control. (TIF) [file pone.0216700.s003.tif]

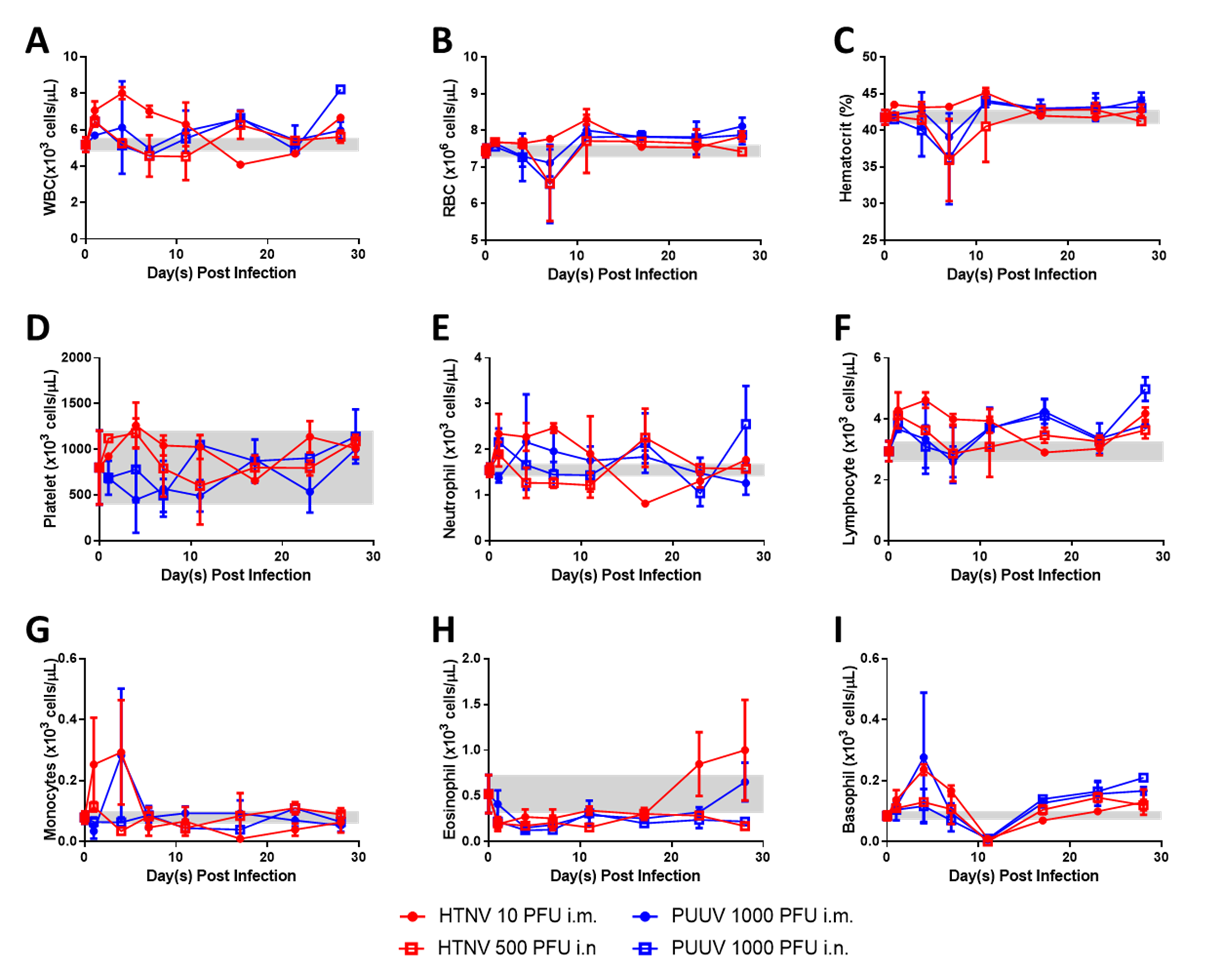

Supplement: S4 Fig — Syrian hamsters were infected either 10 PFU HTNV i.m., 500 PFU HTNV i.n., 1000 PFU PUUV i.m., or 1000 PFU PUUV i.n. Whole blood was collected at the time of euthanasia and evaluated for white blood cell count (A), red blood cell count (B), hematocrit (C), platelets (D), neutrophils (E), lymphocytes (F), monocytes (G), eosinophils (H), basophils (I). The gray box indicates the normal range of hamsters as determined by uninfected control animals. (TIF) [file pone.0216700.s004.tif]

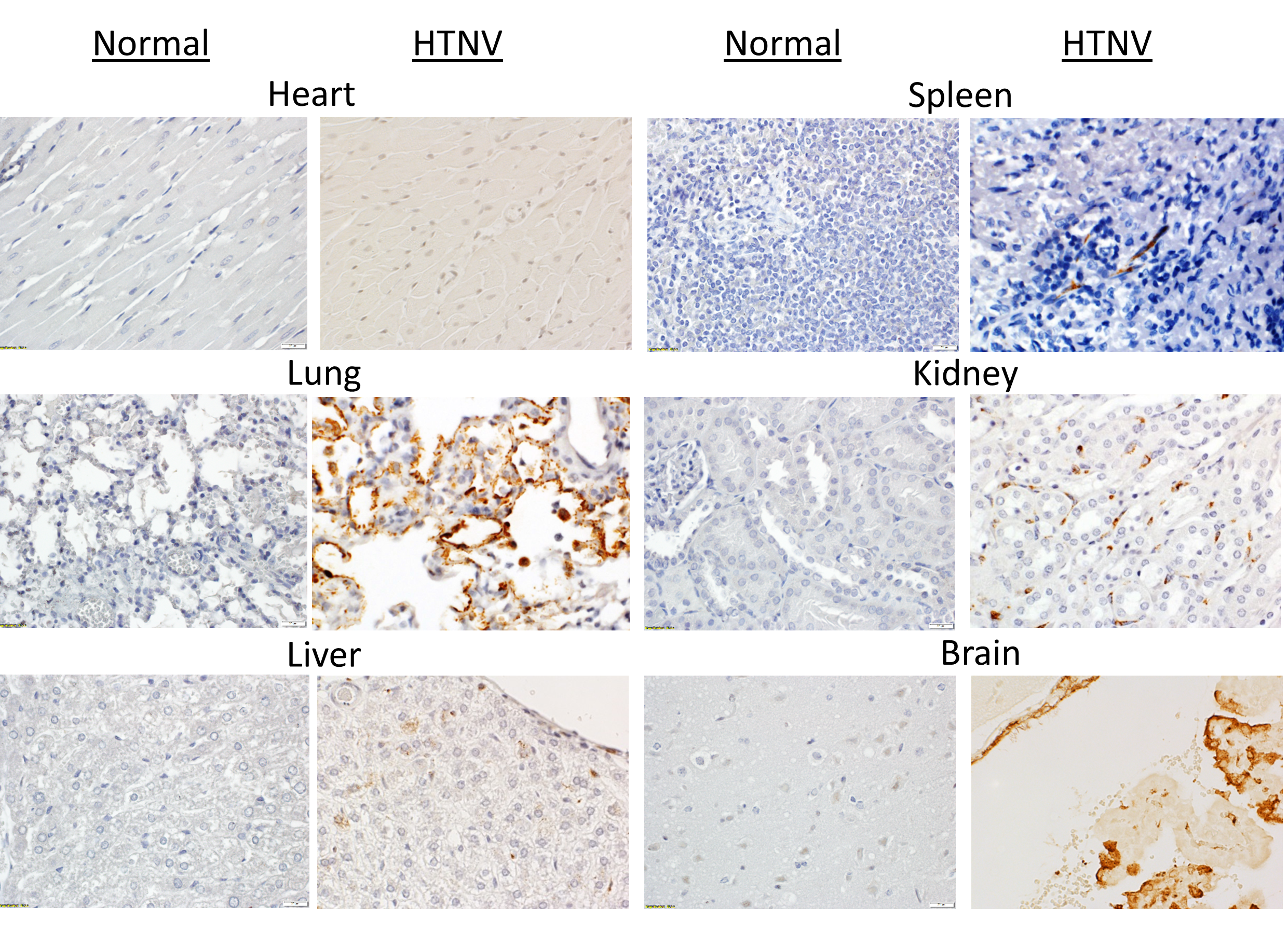

Supplement: S5 Fig — Hamsters were infected with 10 PFU HTNV i.m and sacrificed at various time points post infection. Heart, lung, liver, spleen, kidney and brain tissue were fixed in formalin, sectioned, and stained by IHC to identify HTNV viral antigen. Representative images of organs from normal and Day 28 are shown. Pictures at 400x magnification. (TIF) [file pone.0216700.s005.tif]

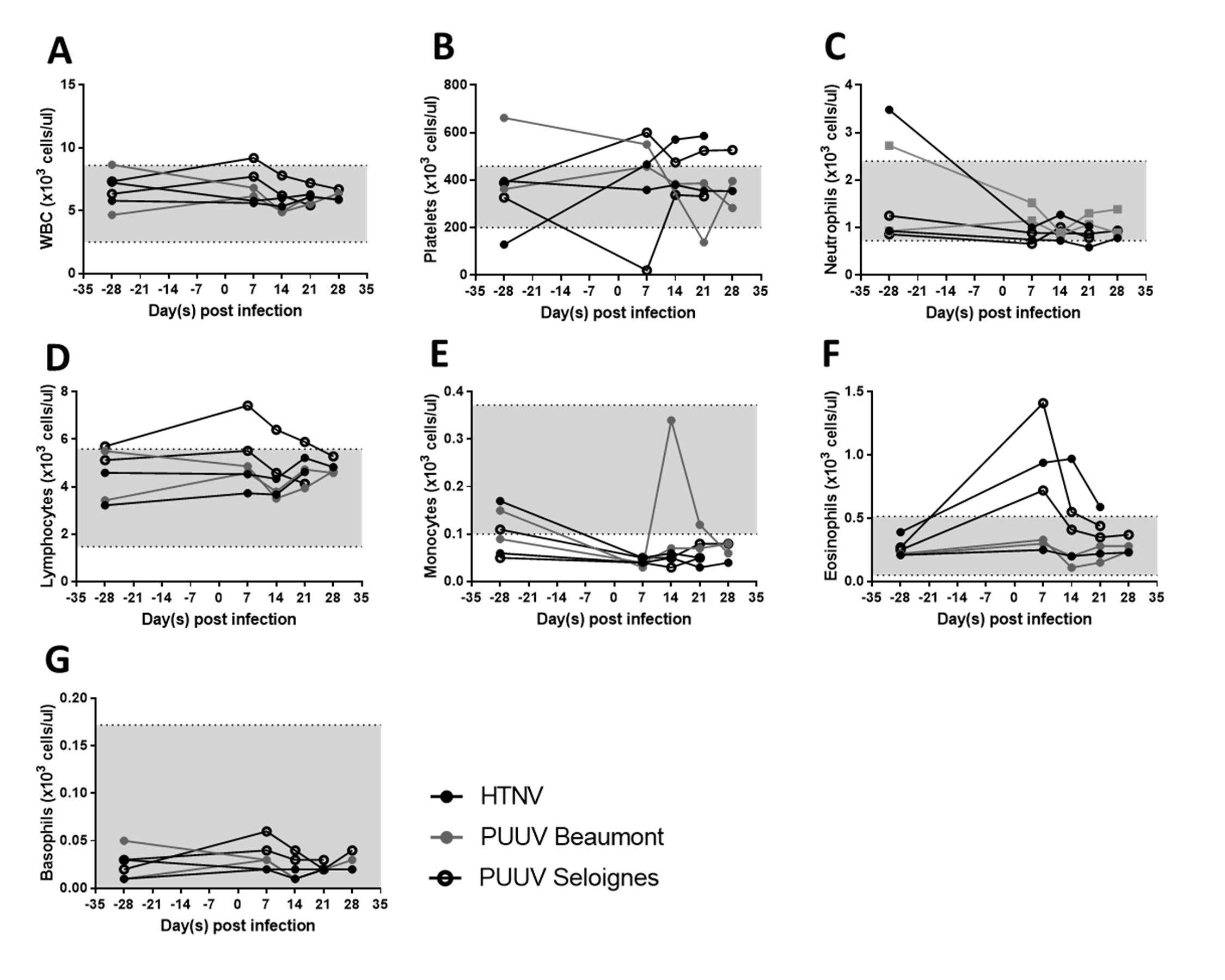

Supplement: S6 Fig — Ferrets were challenged with either 200,000 PFU HTNV, 94,000 PFU PUUV Beaumont, or 164,000 PFU of PUUV Seloignes i.m. Whole blood was drawn weekly post infection and evaluated for White blood cell count (A), platelets (B), neutrophils (C), lymphocytes (D), monocytes (E), eosinophils (F), basophils (G). The gray box represents the average range of values for ferrets [81]. (TIF) [file pone.0216700.s006.tif]

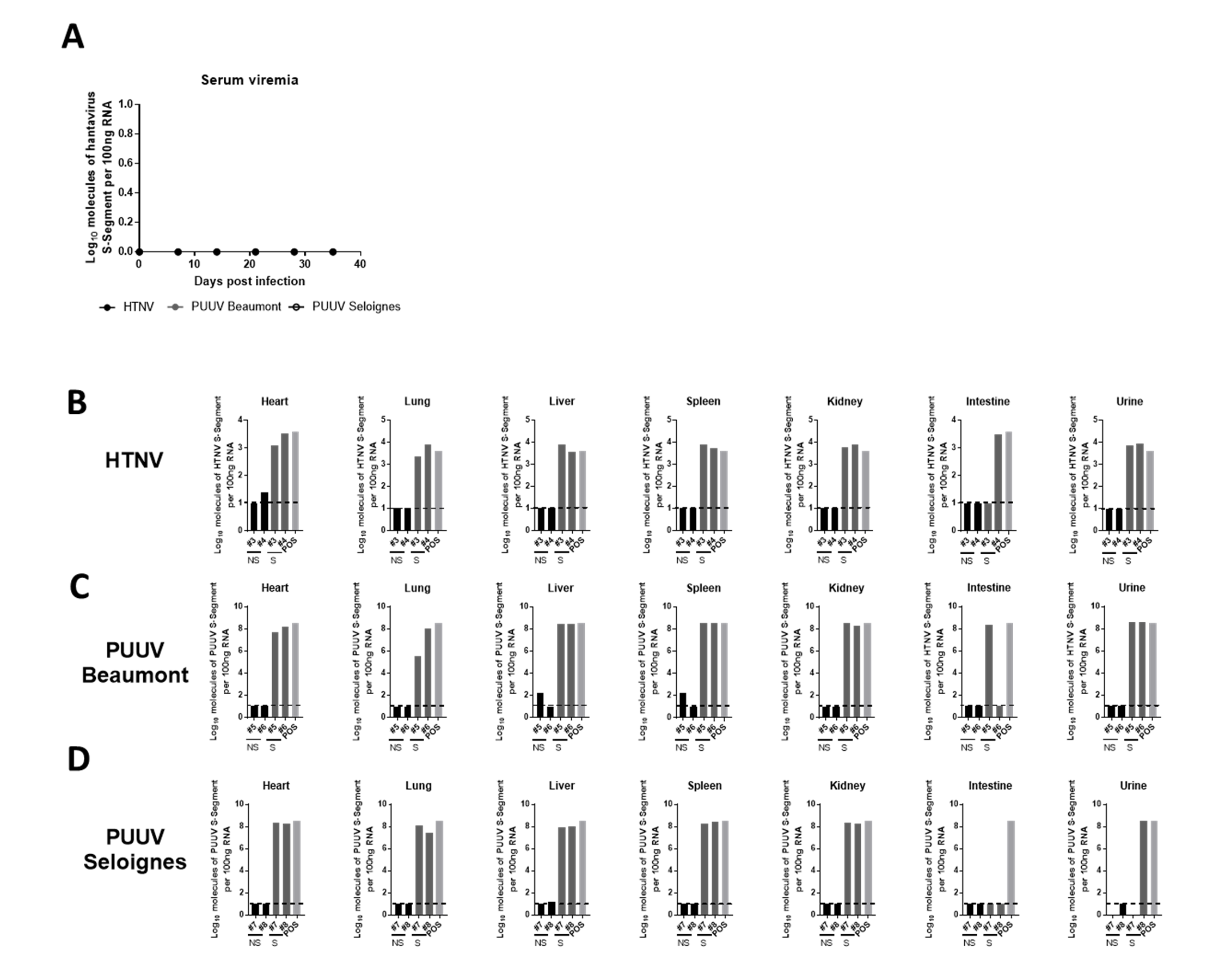

Supplement: S7 Fig — Ferrets were challenged with either 200,000 PFU HTNV, 94,000 PFU PUUV Beaumont, or 164,000 PFU of PUUV Seloignes i.m. on Day 0, and immunosuppressed with 30 mg/kg Cyp on Day 41. Sera was collected weekly and assayed for serum viremia by RT- PCR (A). At time of euthanasia, heart, lung, liver, spleen, kidney, intestine and urine (except #7) were assayed by RT-PCR for the presence of viral genome. No appreciable genome was recovered in ferrets infected with HTNV (B), PUUV Beaumont (C) or PUUV Seloignes (D). Virus was spiked into the samples to confirm no inhibitor was present. The limit of detection for RT-PCR is 1 log10 and is represented by the dashed line. (POS) is virus spiked into water to serve as a control. (TIF) [file pone.0216700.s007.tif]

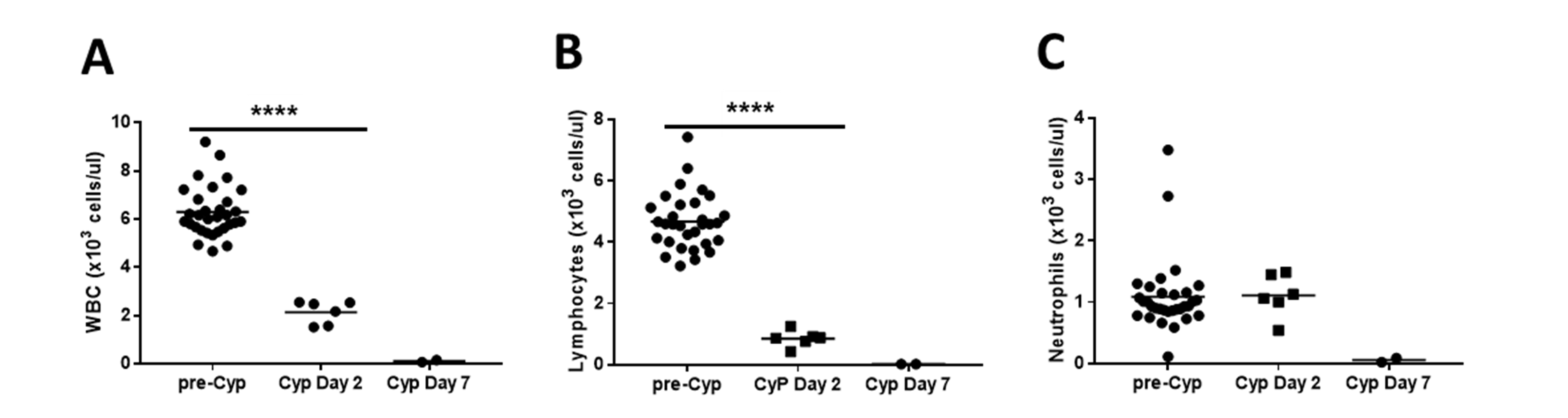

Supplement: S8 Fig — Ferrets were challenged with either 200,000 PFU HTNV, 94,000 PFU PUUV Beaumont, or 164,000 PFU of PUUV Seloignes i.m. and immunosuppressed with 30 mg/kg Cyp every other day beginning on Day 41. Whole blood was drawn from ferrets to evaluate white blood count (WBC) (A), lymphocyte count (B) and neutrophil count (C) prior to, two days post, and seven days post Cyp administration (if alive). Line depicting mean is shown. Student t-test or Mann–Whitney test was used to compare values between pre-Cyp and Cyp day 2 depending on the standard deviation of the groups being compared. As only two ferrets survived until Cyp Day 7 no statistics are possible. **** P<0.0001. (TIF) [file pone.0216700.s008.tif]

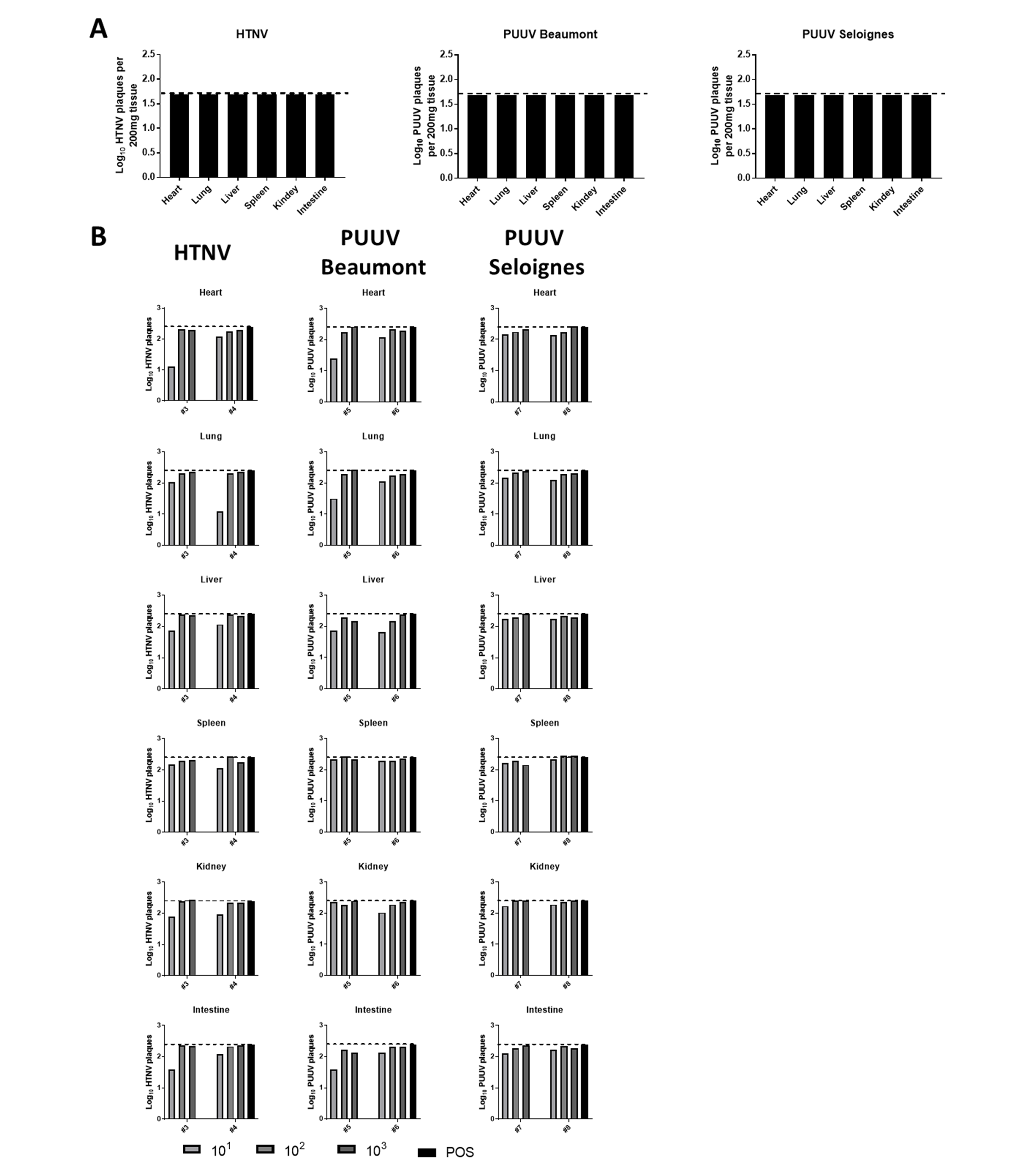

Supplement: S9 Fig — Ferrets were challenged with either 200,000 PFU HTNV, 94,000 PFU PUUV Beaumont, or 164,000 PFU of PUUV Seloignes i.m. on Day 0, and immunosuppressed with 30 mg/kg Cyp on Day 41. Heart, lung, liver, spleen, kidney, and intestine were collected and assayed by plaque assay for the presence of infectious virus (A). To confirm lack of virus recovered was not due to inhibitors, virus was spiked into serial dilutions of organ homogenate to confirm no inhibitor was present (B). For a standard plaque assay the limit of detection, 1.7 log10, is depicted as a dashed line in (A). In (B) the dashed line is amount of HTNV plaques obtained when spiked into media rather than organ homogenate. (TIF) [file pone.0216700.s009.tif]

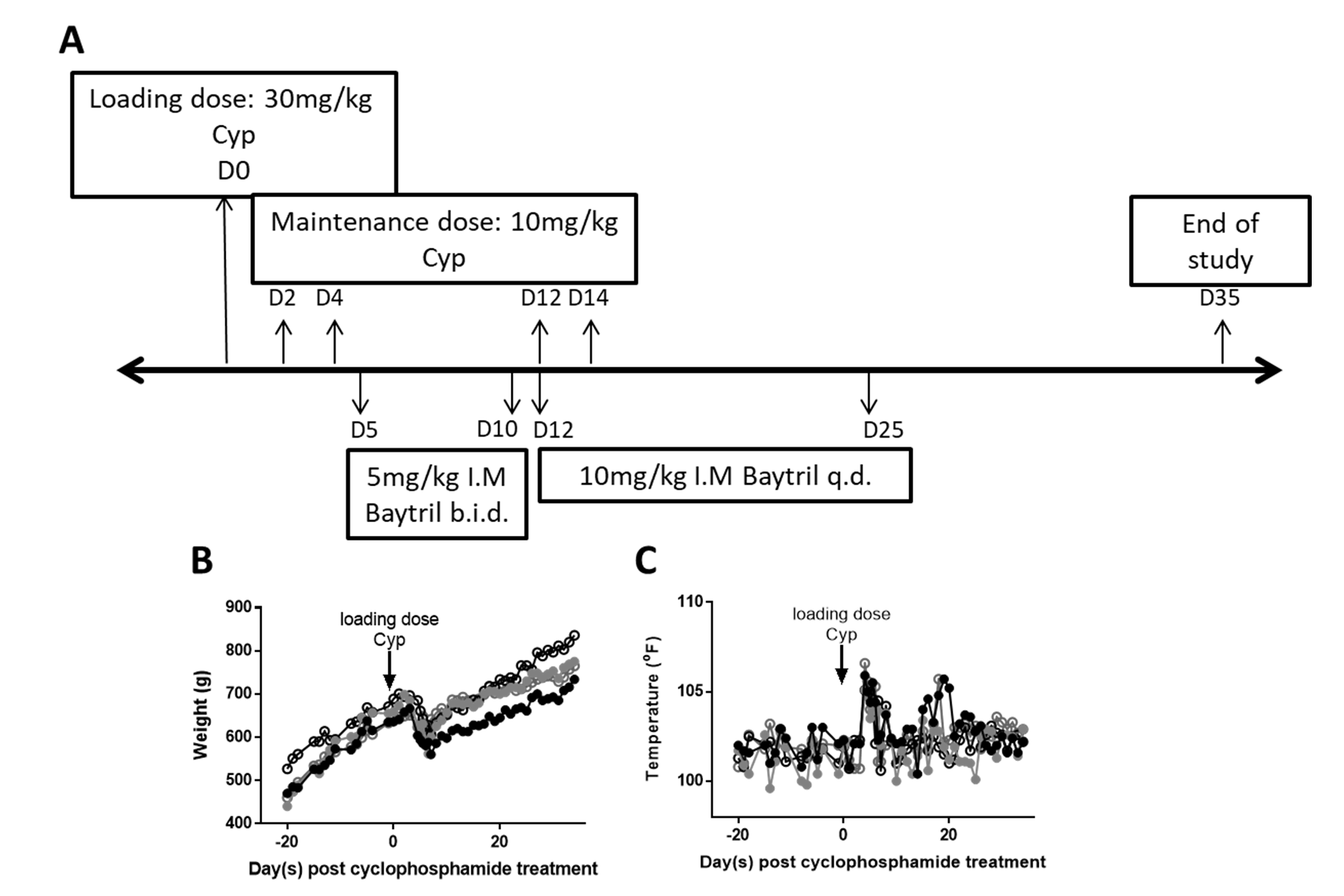

Supplement: S10 Fig — Uninfected ferrets were administered 10mg/kg Cyp, and the antibiotic enrofloxicin, according to the schedule in (A). Weight (B) and temperature (C) are shown. b.i.d indicates antibody was administered twice daily, and q.d indicates antibiotic was administered daily. (TIF) [file pone.0216700.s010.tif]

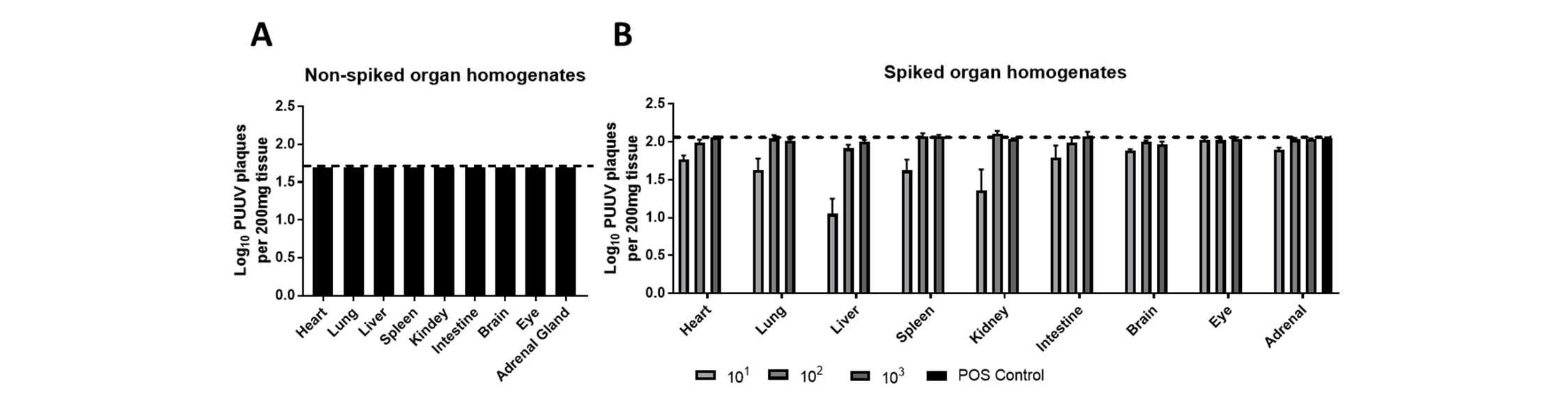

Supplement: S11 Fig — Ferrets were infected with 94,000 PFU PUUV Beaumont i.m. Heart, lung, liver, spleen, kidney, intestine, brain, eye, and adrenal gland were collected on day 35 post infection and assayed for infectious virus by plaque assay (A). Virus was spiked in to confirm no inhi bitor was present (B). For a standard plaque assay the limit of detection, 1.7 log10, is depicted as a dashed line in (A). In (B) the mean ± SEM is depicted in all spiked groups and the dashed line is amount of HTNV plaques obtained when spiked into media rather than organ homogenate. (TIF) [file pone.0216700.s011.tif]

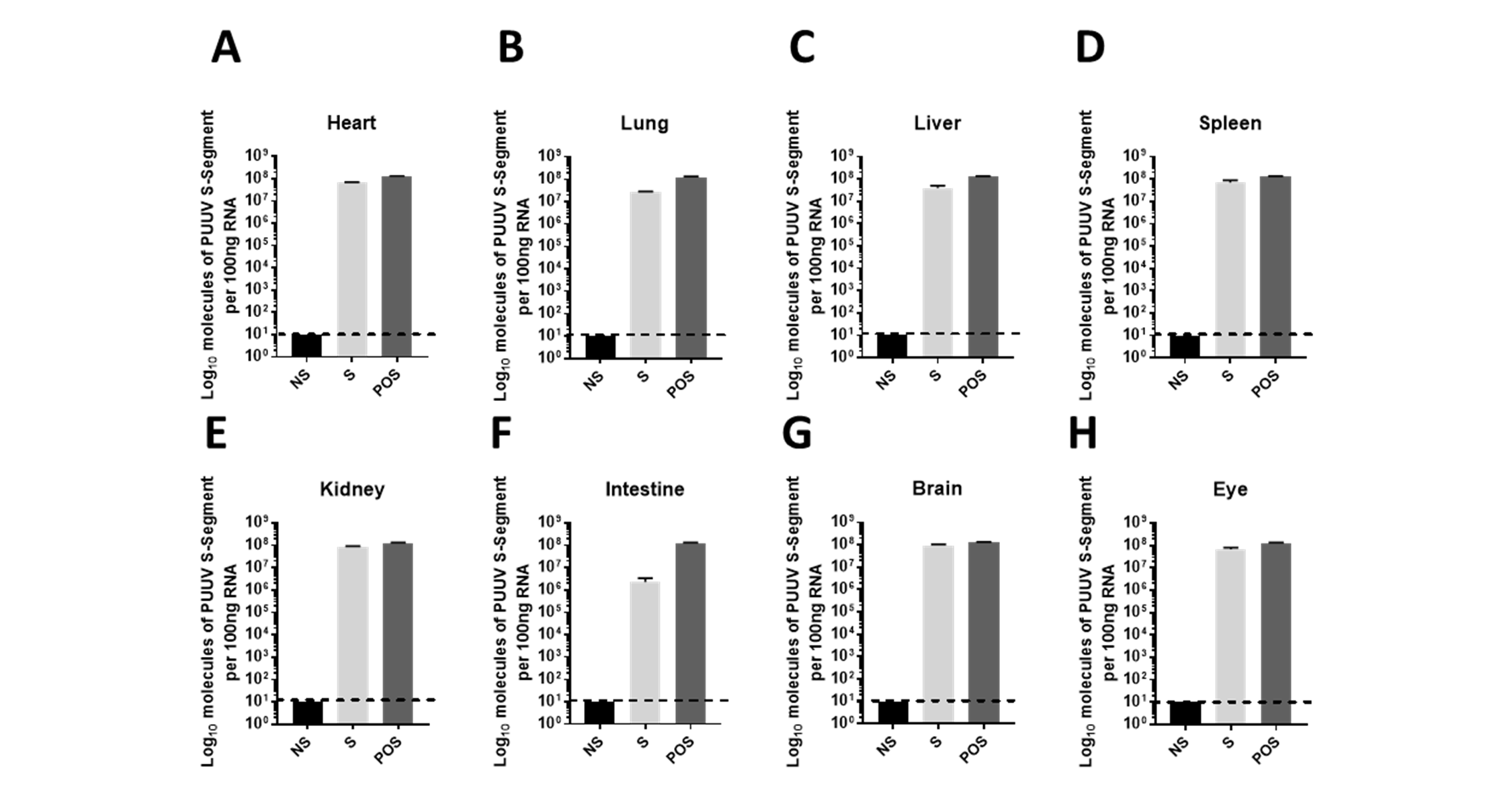

Supplement: S12 Fig — Ferrets were infected with 94,000 PFU PUUV Beaumont i.m. Heart (A), lung (B), liver (C), spleen (D), kidney (E), intestine (F), brain (G), and eye (H), were collected on Day 35 post infection and assayed for viral genome by RT-PCR. Viral genome was spiked in to confirm no inhibitor was present. The mean ± SEM is depicted and the limit of detection, 1 log10, is shown as a dashed line. (NS) not spiked, (S) spiked and (POS) virus spiked into water. (TIF) [file pone.0216700.s012.tif]

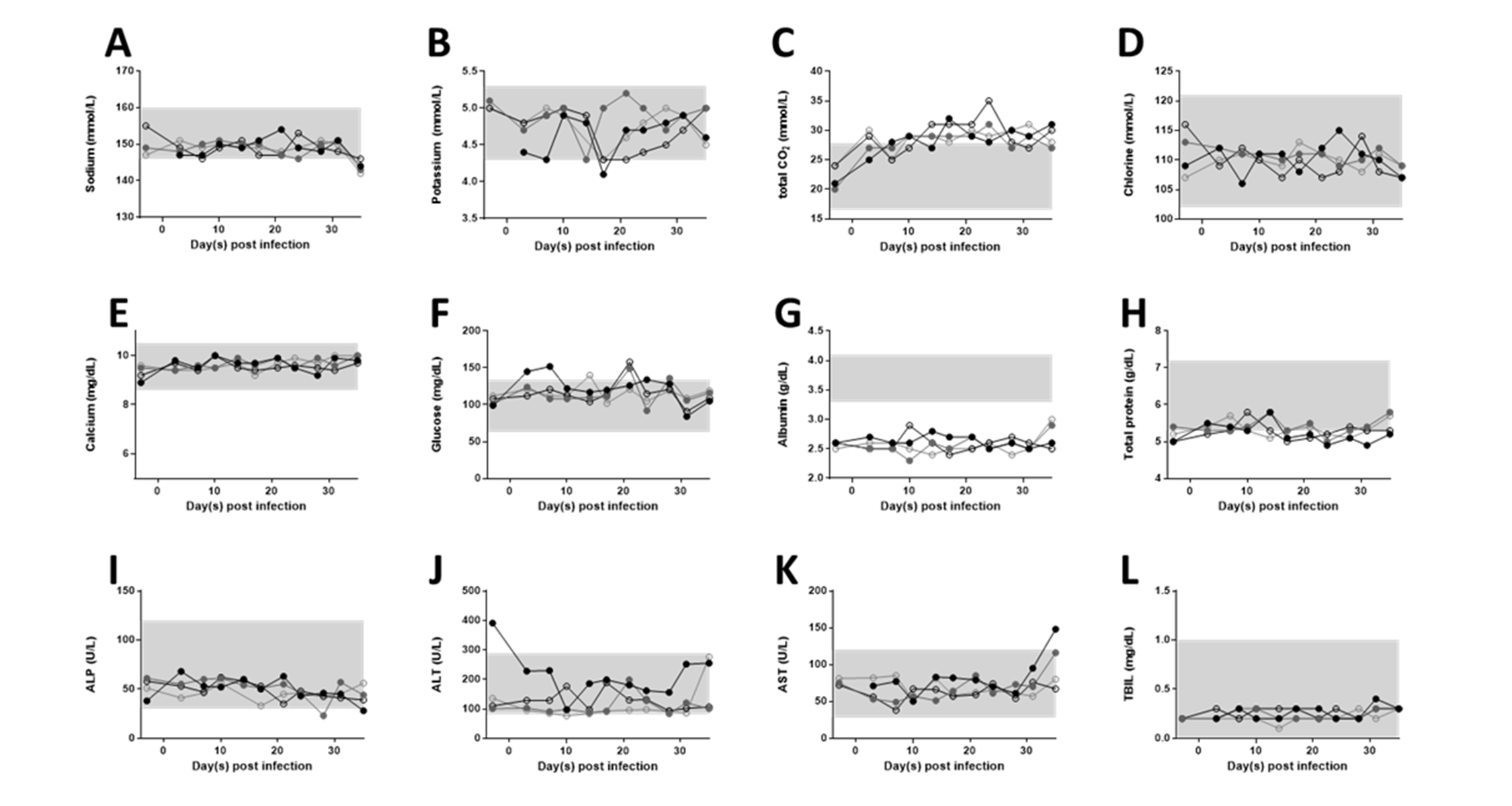

Supplement: S13 Fig — Ferrets were infected with 94,000 PFU PUUV Beaumont i.m. Sera was collected twice weekly for blood chemistry analysis. Sodium (A), potassium (B), total CO2 (C), chlorine (D), calcium (E), glucose (F), albumin (G), total protein (H), ALP (I), ALT (J), AST (K), total bilirubin (L). Shaded gray areas represent normal range (all reference values except tC02 from [48], tC02 from [49]). (TIF) [file pone.0216700.s013.tif]

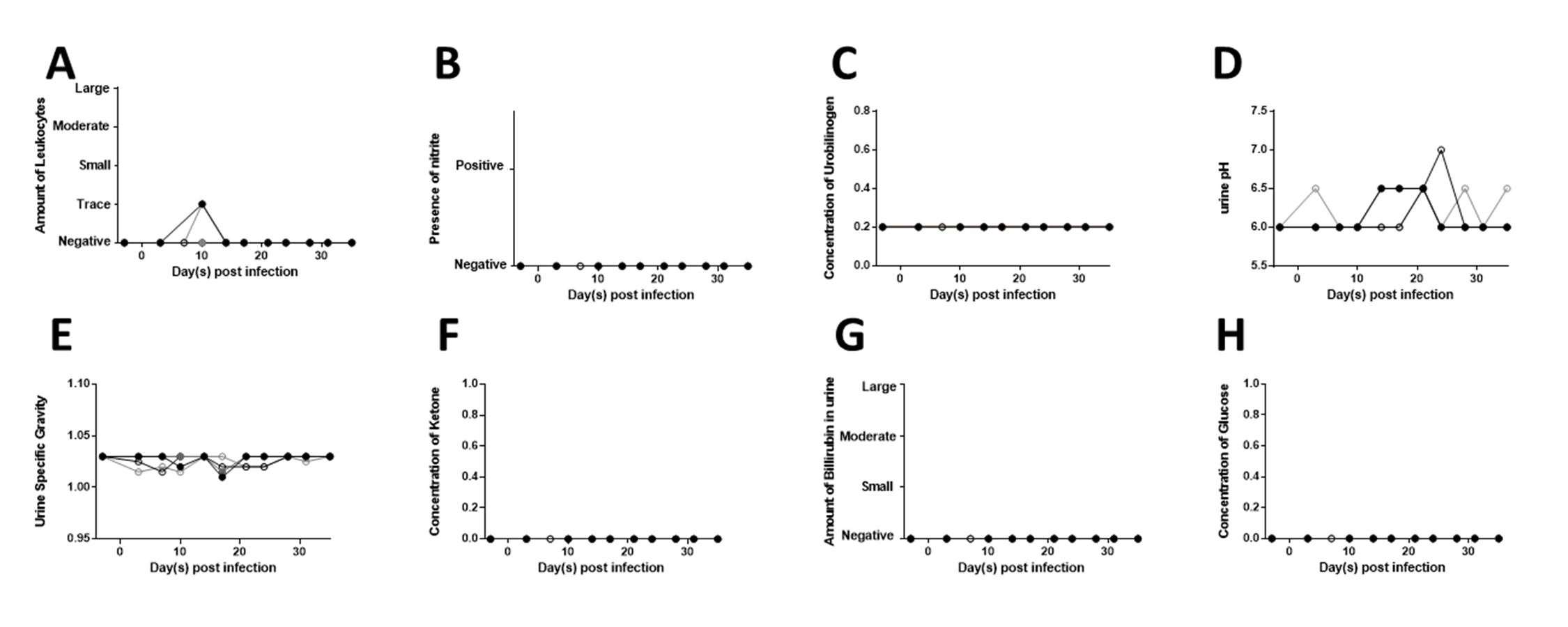

Supplement: S14 Fig — Ferrets were infected with 94,000 PFU PUUV Beaumont i.m. Urine was collected twice weekly for urinalysis. Leukocytes (A), nitrite (B), urobilinogen (C), pH (D), specific gravity (E), ketone (F), bilirubin (G), and glucose (H). (TIF) [file pone.0216700.s014.tif]

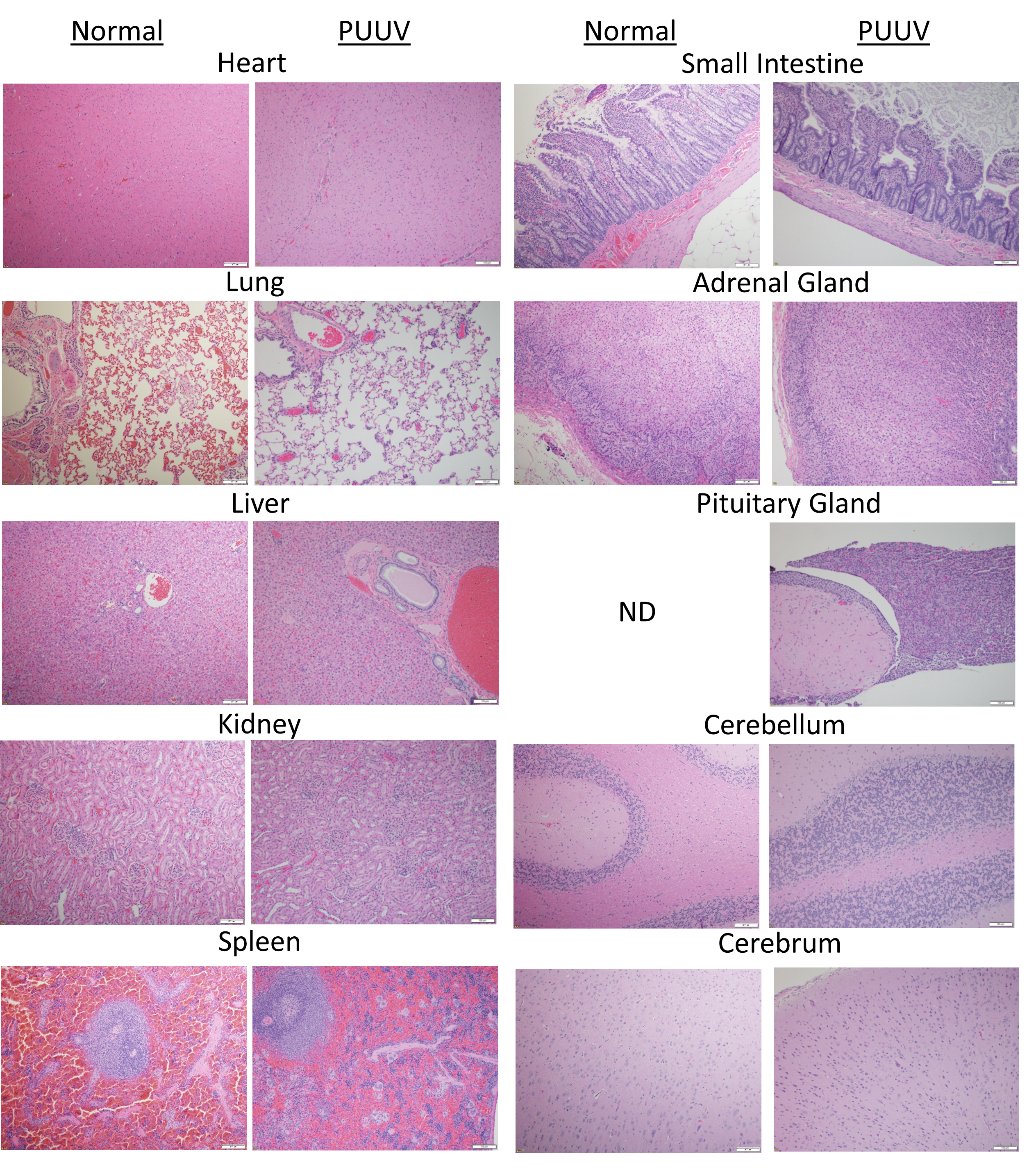

Supplement: S15 Fig — Ferrets were euthanized 35 days post a 94,000 PUUV Beaumont i.m. challenge. Heart, lung, liver, kidney, spleen, small intestine, adrenal gland, pituitary gland, cerebellum and cerebrum were evaluated by H&E. Pictures taken at 10x magnification. ND, no data. (TIF) [file pone.0216700.s015.tif]

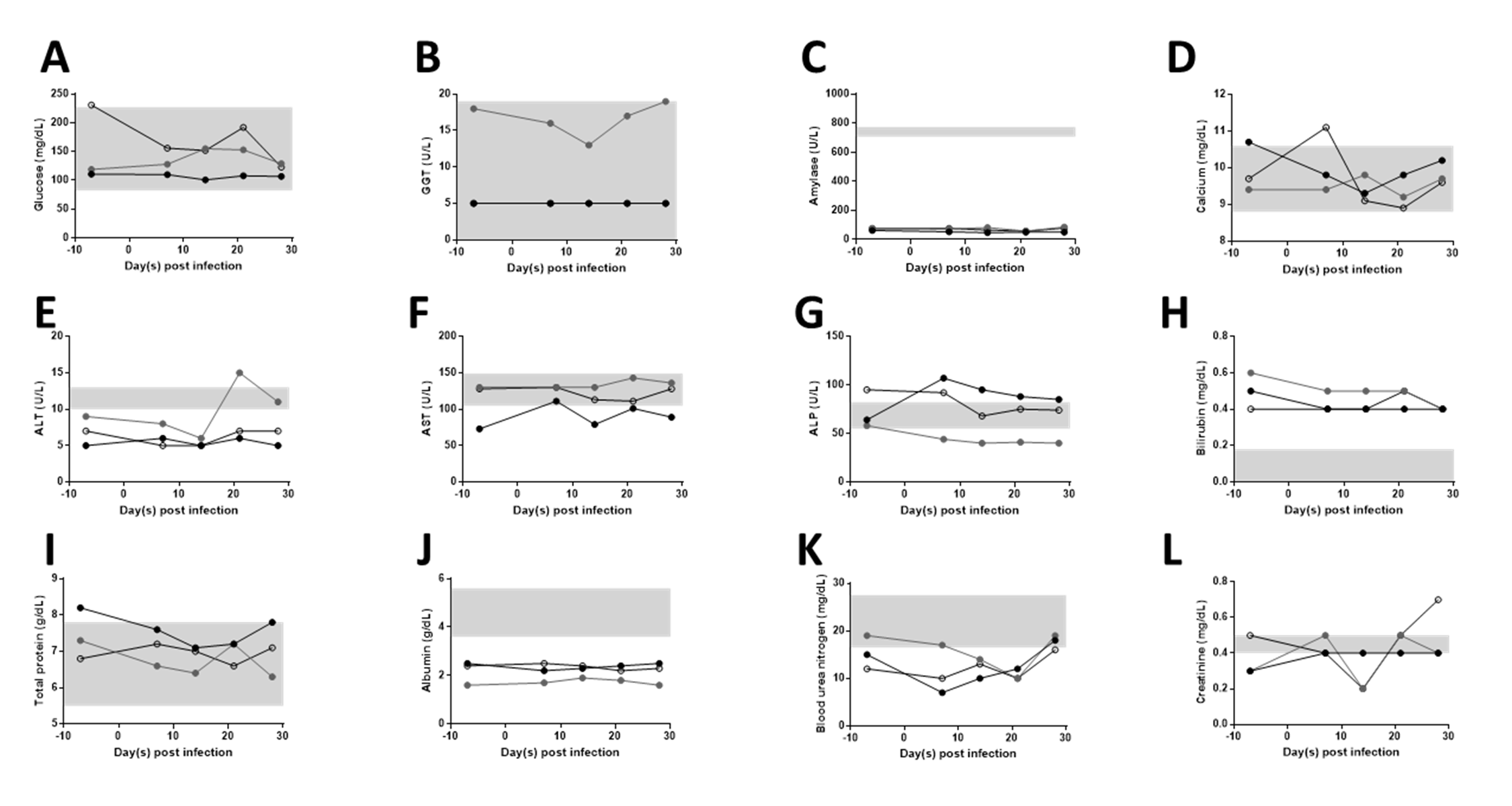

Supplement: S16 Fig — Three marmosets were infected with 1,000 PFU HTNV i.m. Sera was collected weekly for blood chemistry analysis. Glucose (A), GGT (B), amylase (C), calcium (D), ALT (E), AST (F), ALP (G), total bilirubin (H), total protein (I) albumin (J), blood urea nitrogen (K), and creatinine (L) were measured. Gray shaded area represents reference values (all reference values except GGT and bilirubin from [79], reference values for GGT and bilirubin from [80]). (TIF) [file pone.0216700.s016.tif]

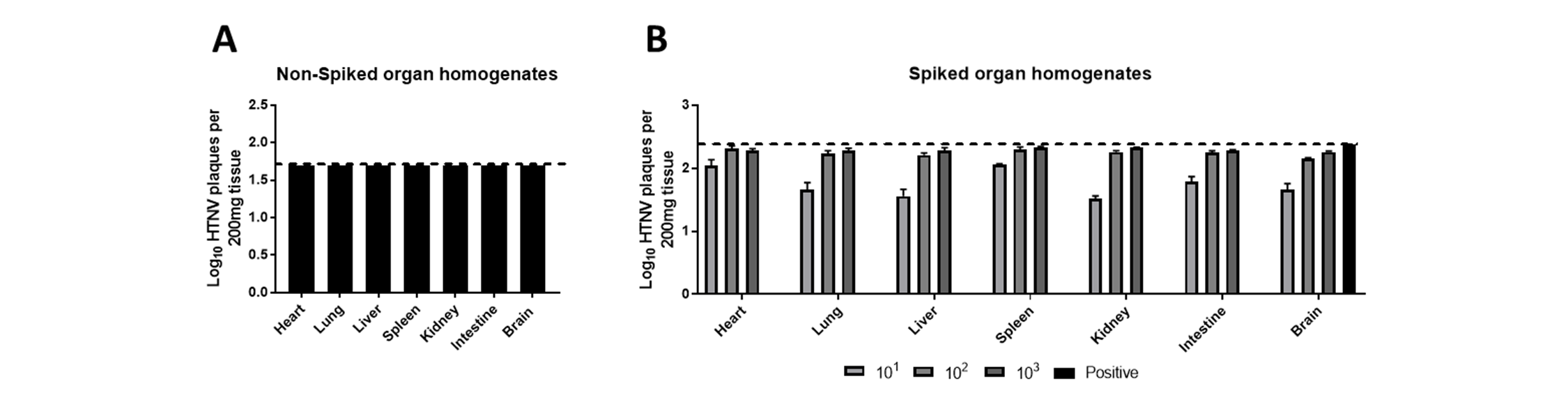

Supplement: S17 Fig — Three marmosets were infected with 1,000 PFU HTNV i.m. On Day 30 post infection organs were harvested, and the presence of infectious virus was determined by plaque assay (A). To confirm no inhibitors were present, virus was spiked into samples (B). For a standard plaque assay the limit of detection, 1.7 log10, is depicted as a dashed line in (A). In (B) the mean ± SEM is displayed for all spiked groups, and the dashed line is amount of HTNV plaques obtained when spiked into media rather than organ homogenate. (TIF) [file pone.0216700.s017.tif]

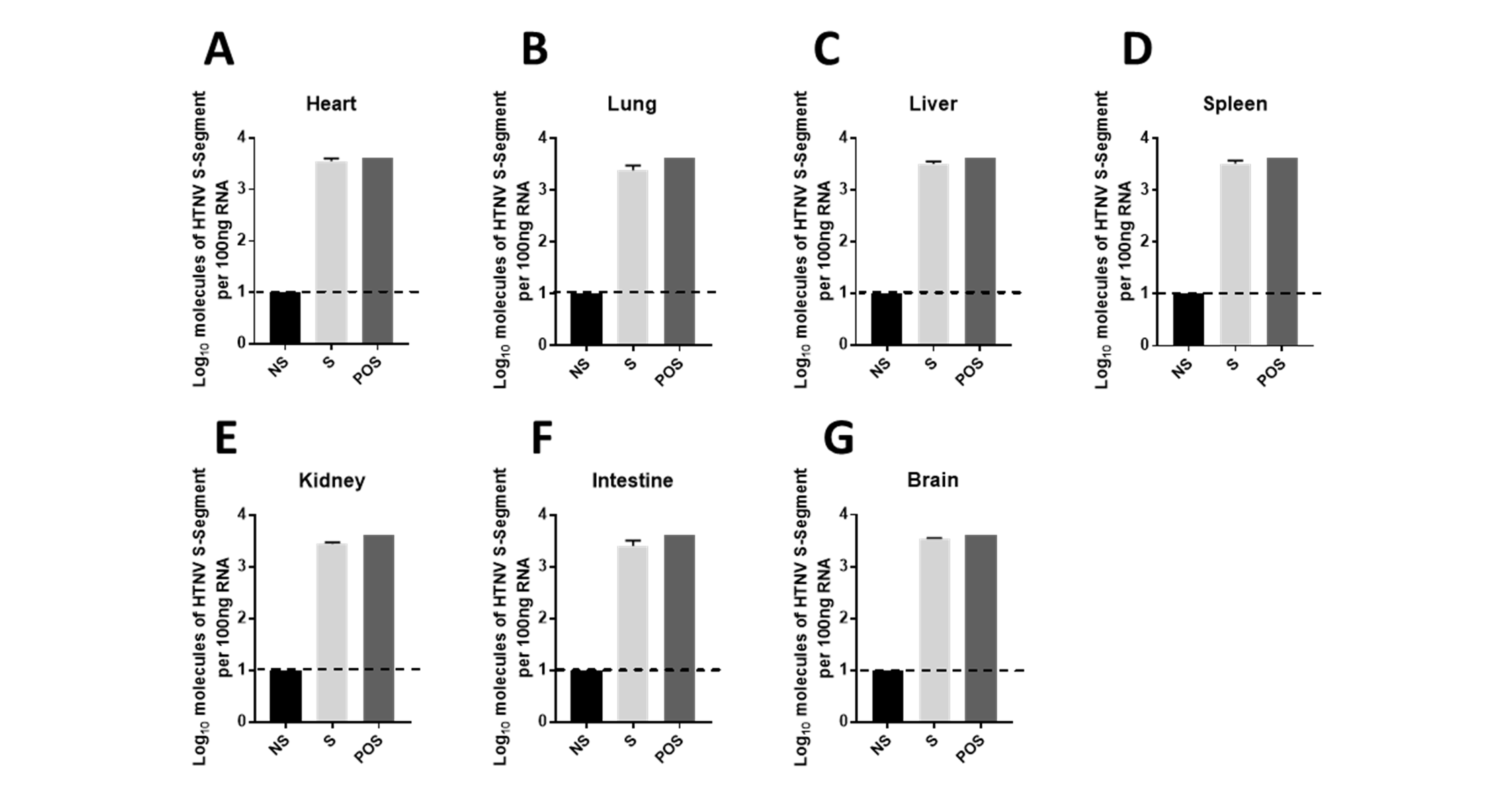

Supplement: S18 Fig — Three marmosets were infected with 1,000 PFU HTNV i.m. On Day 30 organs were harvested, and the presence of viral genome was determined by RT-PCR. To confirm no inhibitors were present, viral genome was spiked into samples. Heart (A), lung (B), liver (C), spleen (D), kidney (E), intestine (F), and brain (G) were collected. The mean ± SEM is shown for the not spiked (NS) and spiked (S) groups and the limit of detection, 1 log10, is depicted as a dashed line. (POS) is viral genome spiked into water. (TIF) [file pone.0216700.s018.tif]

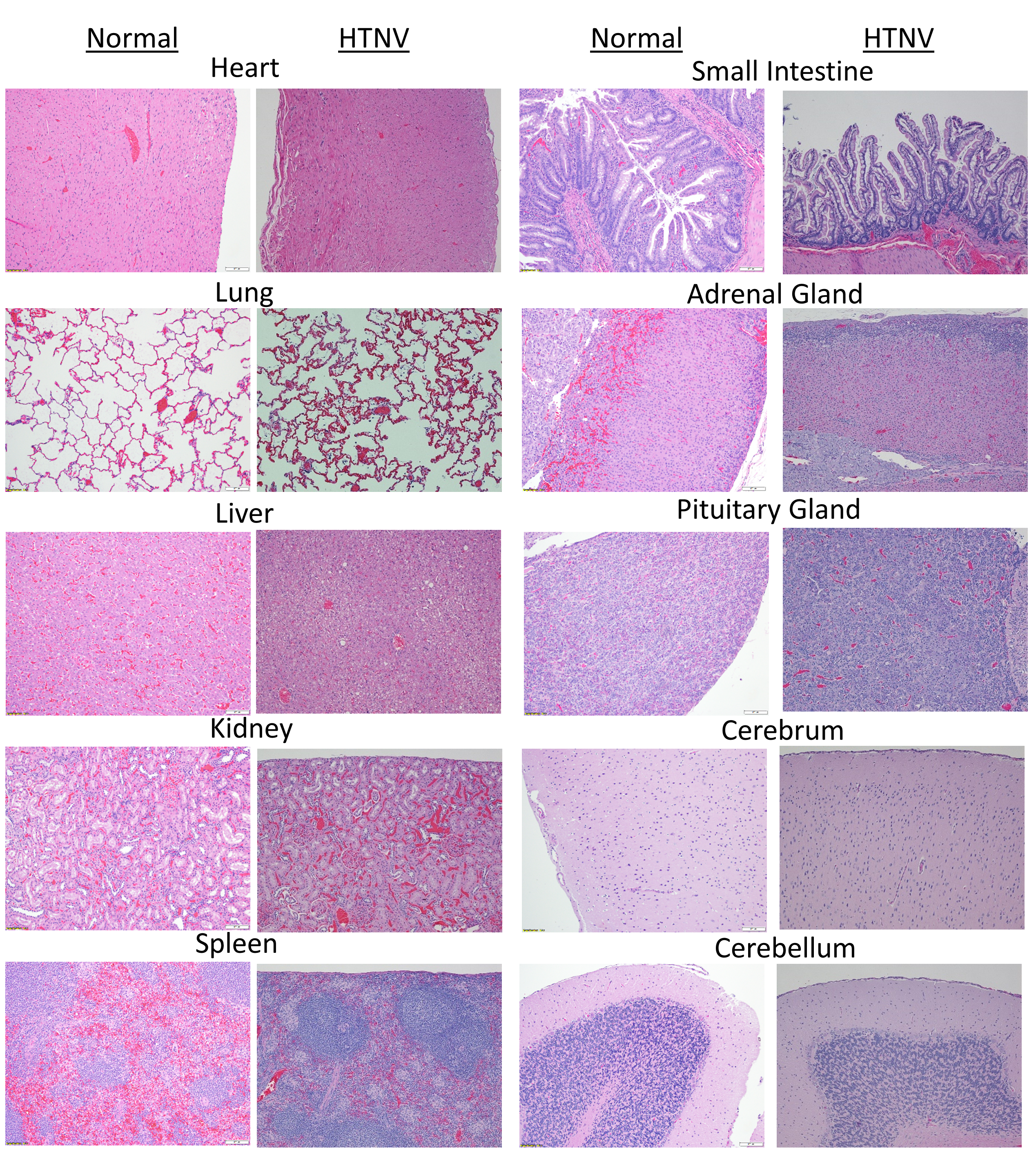

Supplement: S19 Fig — Marmosets were euthanized 30 days post 1,000 PFU HTNV i.m. challenge. Heart, lung, liver, kidney, spleen, small intestine, adrenal gland, pituitary gland, cerebrum, and cerebellum from normal and infected animals were stained by H&E for gross pathological changes. Representative images in this Fig are taken from all three animals. Pictures at 10x magnification. (TIF) [file pone.0216700.s019.tif]
